# Development of a machine learning model to predict overall survival for large hepatocellular carcinoma at BCLC stage A or B after curative hepatectomy

**DOI:** 10.3389/fimmu.2025.1640075

**Published:** 2025-10-21

**Authors:** Tai-Xin Yang, Jia-Yong Su, Min-Jun Li, Shuang Shen, Yu Wang, Huan-Nan Wei, Ming-Jian Huang, Qing-Man Qin, You-Yin Ran, Yao-Ting Huang, Jin-Yan Huang, Bang-De Xiang, Jie Zhang, Wen-Feng Gong

**Affiliations:** ^1^ Department of Hepatobiliary Surgery, Guangxi Medical University Cancer Hospital, Nanning, Guangxi, China; ^2^ Guangxi Medical University, , Nanning, China; ^3^ Key Laboratory of Early Prevention and Treatment for Regional High Frequency Tumors, Guangxi Medical University, Ministry of Education, Nanning, China; ^4^ Guangxi Key Laboratory of Early Prevention and Treatment for Regional High Frequency Tumors, Nanning, China

**Keywords:** gradient boosting machine, hepatectomy, large hepatocellular carcinoma, overall survival, SHAP

## Abstract

**Introduction:**

Patients with large hepatocellular carcinoma (LHCC) have a poor prognosis even after curative hepatectomy. This study aimed to develop and validate an interpretable machine learning (ML) model to predict their overall survival (OS).

**Methods:**

This study included 2,565 patients with hepatocellular carcinoma (HCC) who underwent curative hepatectomy between January 2014 and December 2021. The LHCC patients were randomly assigned (7:3 ratio) to a training (n=1069) or validation (n=457) group. Independent risk factors for OS were identified using multivariable Cox regression. Eight ML models were developed and compared. The optimal model’s interpretability was assessed using Shapley Additive Explanations (SHAP).

**Results:**

LHCC patients experienced a considerable reduction in OS (Hazard Ratio, HR: 1.810, 95% Confidence Interval, CI: 1.585-2.068) compared to SHCC patients. Among eight ML models, the gradient boosting machine (GBM) model demonstrated superior performance. In the validation group, the GBM model achieved area under the receiver operating characteristic curve (AUC) values of 0.742, 0.744, and 0.750 for 1-, 3-, and 5-year OS, respectively. These results were comparable with or superior to established postoperative predictive models. The GBM model showed the ability to stratify patients with LHCC into distinct prognostic groups. A web-based calculator was developed for risk score generation. Notably, the GBM model showed enhanced predictive accuracy in patients with a high neutrophil-lymphocyte ratio (C-index: 0.819).

**Conclusions:**

The GBM-based model demonstrated the potential to predict prognosis for patients with LHCC after curative hepatectomy. This interpretable model may assist in personalized risk assessment and tailoring postoperative management strategies.

## 1 Introduction

Across the globe, hepatocellular carcinoma (HCC) is the third leading cause of death related to cancer, with many patients receiving a diagnosis after tumors have reached an advanced size (1, 2). Despite advancements in treatment modalities—including hepatectomy, liver transplantation, local ablation, targeted therapy, and immunotherapy—the prognosis for patients with large hepatocellular carcinoma (LHCC) remains poor, characterized by low 5-year survival rates (3–7). This grim outlook is largely attributed to the higher risk of microvascular invasion (MVI) associated with LHCC, a critical oncological factor linked to unfavorable outcomes (8, 9).

Accurate prognosis predictions for LHCC patients enable medical professionals to design individualized treatment strategies, assess survival risks, and enhance the overall quality of life for patients. In clinical practice, the Barcelona Clinic Liver Cancer (BCLC) staging system stands as a commonly employed approach for liver cancer, but it inadequately addresses the complex variations in individual patient factors and tumor malignancy behaviors (10). Machine learning (ML) technology is rapidly evolving and is increasingly applied in the medical field. ML can potentially analyze complex datasets, uncover hidden patterns, and derive insights that could pave the way for novel approaches to tumor prognostication (11–14).

In recent years, ML has demonstrated considerable advantages in predicting HCC prognosis by analyzing multidimensional clinical information. However, the “black-box” nature of ML models presents challenges for clinical practice. To overcome these obstacles, the explainable artificial intelligence emerges as a reliable tactic to interpret ML models’ outputs and elucidate the derivation process of these models. This transparency is crucial for clinicians to trust and effectively integrate ML models into their practice (15–19). In this study, we utilize the ML models in combination with the Shapley Additive Explanations (SHAP) explainability framework to stratify patients with LHCC and assist in treatment decisions (20–23).

## 2 Materials and methods

### 2.1 Patients

The investigation focused on HCC patients who received curative hepatectomy at Guangxi Medical University Cancer Hospital from January 2014 through December 2021. Curative hepatectomy was defined as an R0 resection, with no microscopic tumor cells at the surgical margin, according to the Diagnosis and Treatment Guidelines for Primary Liver Cancer (24); The study’s criteria for participant selection were defined by specific inclusion and exclusion parameters. Inclusion criteria included: (1) patients were underwent R0 resection and all enrolled patients had adequate liver function reserve, as defined by an indocyanine green 15-minute retention rate (ICG R15) ≤30%; (2) Child–Pugh score of 5-7; (3) Eastern Cooperative Oncology Group performance status (ECOG PS) of 0 or 1; and (4) BCLC stage A or B. Exclusion criteria comprised: (1) history of other malignancies, (2) any preoperative anticancer treatment such as adjuvant chemotherapy, targeted therapy, immunotherapy, interventional therapy, or radiotherapy; (3) postoperative therapy, including aforementioned treatments; and (4) incomplete clinical data and follow-up duration of less than 2 months.

The Guangxi Medical University Ethics Committee (KY2025413) approved this study, which adhered to the Declaration of Helsinki principles.

### 2.2 Clinicopathologic variables and follow-up

Clinicopathological information of patients with HCC were collected, including (1) demographic information: gender, age, height, weight, etc. (2) laboratory parameters: total bilirubin, alpha-fetoprotein (AFP), albumin, platelets, etc. (3) liver disease-related information: Hepatitis B virus (HBV) infection status, HBV DNA level, etc. (4) tumor-related information: tumor number, tumor size, postoperative pathology, etc. The HCC stage was evaluated according to the BCLC staging classification system (25).

The main purpose was to assess overall survival (OS), tracked from the date of curative hepatectomy to either death from any cause or the last follow-up. The secondary endpoint was recurrence-free survival (RFS), defined as the time from curative hepatectomy until to the first occurrence of either disease recurrence or death from any cause.

Postoperative follow-ups were conducted at intervals of 1–2 months for the first year, followed by every 3 months thereafter until recurrence occurred. The follow-up programs included regular evaluations of liver function, AFP levels, and at least one contrast-enhanced imaging. HCC recurrence was diagnosed through a thorough evaluation of clinical history, AFP tests, and imaging results. The follow-up continued until 26 January 2025.

### 2.3 Statistical analysis

Continuous variables were expressed as means along with standard deviations (SD) and analyzed via Student’s t-test. Alternatively, they were presented as medians together with interquartile ranges (IQR) and analyzed using the Mann–Whitney U test. Categorical variables were presented as n (%) and compared with the Chi-square test. The Kaplan-Meier method was employed to generate the OS curves, and the log-rank test was utilized for their analysis. A multivariable Cox regression analysis model was developed to estimate the likelihood of hepatectomy risk, incorporating predictive factors identified through univariate analysis.

The dataset used for this analysis was complete, with no missing values for the analyzed variables. To develop and validate the predictive models, the entire dataset was randomly split into a training group (70%) and an internal validation group (30%) using the createDataPartition function from the caret package in R. This function employs a stratified random sampling strategy based on the OS status to ensure an equal distribution of deaths between the training and validation sets, thereby improving the robustness of the model evaluation. The random seed was set to 123 to ensure the complete reproducibility of the data partitioning.

Univariate Cox regression analyses were first performed to identify potential prognostic factors. To control the false discovery rate resulting from multiple testing, the p-values from the univariate analysis were further adjusted using the False Discovery Rate (FDR) correction via the Benjamini-Hochberg method. Variables with an FDR-adjusted p-value (P_FDR) < 0.05 were considered statistically significant and selected for inclusion in the subsequent multivariate Cox regression analysis. The proportional hazards assumption for the final multivariate model was verified using Schoenfeld residual tests, and no significant violations were found (global test p >0.05). Multicollinearity among the covariates in the multivariate Cox regression was assessed using the variance inflation factor (VIF). All VIF values were well below the threshold of 5 (BCLC: 1.23, MVI: 1.15, Size: 1.08), indicating no severe multicollinearity that would adversely affect the model estimates.

The independent risk factors identified from the multivariate Cox regression (BCLC stage, MVI, and tumor size) were used as input features for constructing eight ML models. These models include least absolute shrinkage and selection operator regression (Lasso_Cox), gradient boosting machine (GBM), random survival forests (RSF), boosting for Cox’s proportional hazards model (Coxboost), survival support vector machine (Survivalsvm), extreme gradient boosting (xgboost), super-predictor Cox model (superpc), and partial least squares with Cox’s proportional hazards model (plsRcox). The details of the algorithms and their hyperparameters are summarized in **Supplementary Table S1** and **Supplementary Figure S1**.

Hyperparameter tuning is a critical step to optimize model performance and prevent overfitting. For all models, hyperparameter tuning was conducted exclusively on the training group using resampling methods to avoid any information leakage. The specific tuning strategy, search spaces for key hyperparameters, and the criterion for evaluating model performance are described in detail for each algorithm in **Supplementary Table S1** and **Supplementary Figure S1**. We employed a systematic approach based on K-fold cross-validation (with K = 10) for hyperparameter exploration. The internal validation for hyperparameter tuning was performed using stratified 10-fold cross-validation (CV) on the training set. The stratification was based on the OS status to maintain the proportion of events (deaths) consistent across all folds. The performance of each hyperparameter combination was evaluated using the concordance index (C-index). The model configurations identified through this CV process were then evaluated on the independent internal validation set. The final optimal hyperparameter configuration for each algorithm was selected based on the highest average C-index across the 10 stratified CV folds. Critically, the independent internal validation set (30% of the data, held out from all tuning processes) was used only for post-selection evaluation of generalizability to unseen data—this strict separation ensures no information leakage into model selection. The mean and standard deviation of the C-index from both the 10-fold CV process and the independent internal validation for each final model are reported in **Supplementary Table S2**.

The final model for each algorithm, with its hyperparameters fixed to the optimized values, was then refit on the entire training group and subsequently applied to the held-out validation group for an unbiased assessment of its performance. The performance of the ML models was comprehensively assessed using multiple metrics: the C-index, the area under the receiver operating characteristic curve (AUC), calibration curves, decision curve analysis (DCA), the Integrated Brier Score (IBS), and the Net Reclassification Index (NRI). In this study, a Cox proportional hazards model containing no predictor variables (the Null Model) was selected as the reference for NRI calculation. This model represents the average risk of the entire study cohort. This setup allows us to evaluate the absolute incremental value of all ML models over a “no-information” baseline. The NRI was calculated on the training set at 1-, 3-, and 5-year post-hepatectomy. The risk stratification threshold for all models was set at the median of their predicted risk probabilities.

The GBM model was also further compared with previously reported predictive models, including the BCLC staging system, metroticket Cox regression, Tumor-burden score (TBS), ERASL-pre score, and ERASL-post score, using similar evaluation metrics. These comparator models were applied based on their original published algorithms and were not re-trained on our dataset. Patients diagnosed with LHCC were divided into high-risk and low-risk groups based on the median risk scores obtained from the ML models.

The shapviz package was used to visualize contributions in the GBM model. SHAP summary plots showed feature impacts on predictions. Higher SHAP values in the GBM model meant a higher death likelihood. The SHAP feature importance plot orders features based on their average absolute SHAP values. SHAP force plots used colors (orange for positive, dark red for negative) to denote feature contributions.

For two-tailed tests, statistical significance was defined as a p-value of less than 0.05. All the statistical analyses were carried out using R version 4.4.2 (http://www.r-project.org/).

## 3 Results

### 3.1 Postoperative prognosis of patients with HCC

**Figure 1:**
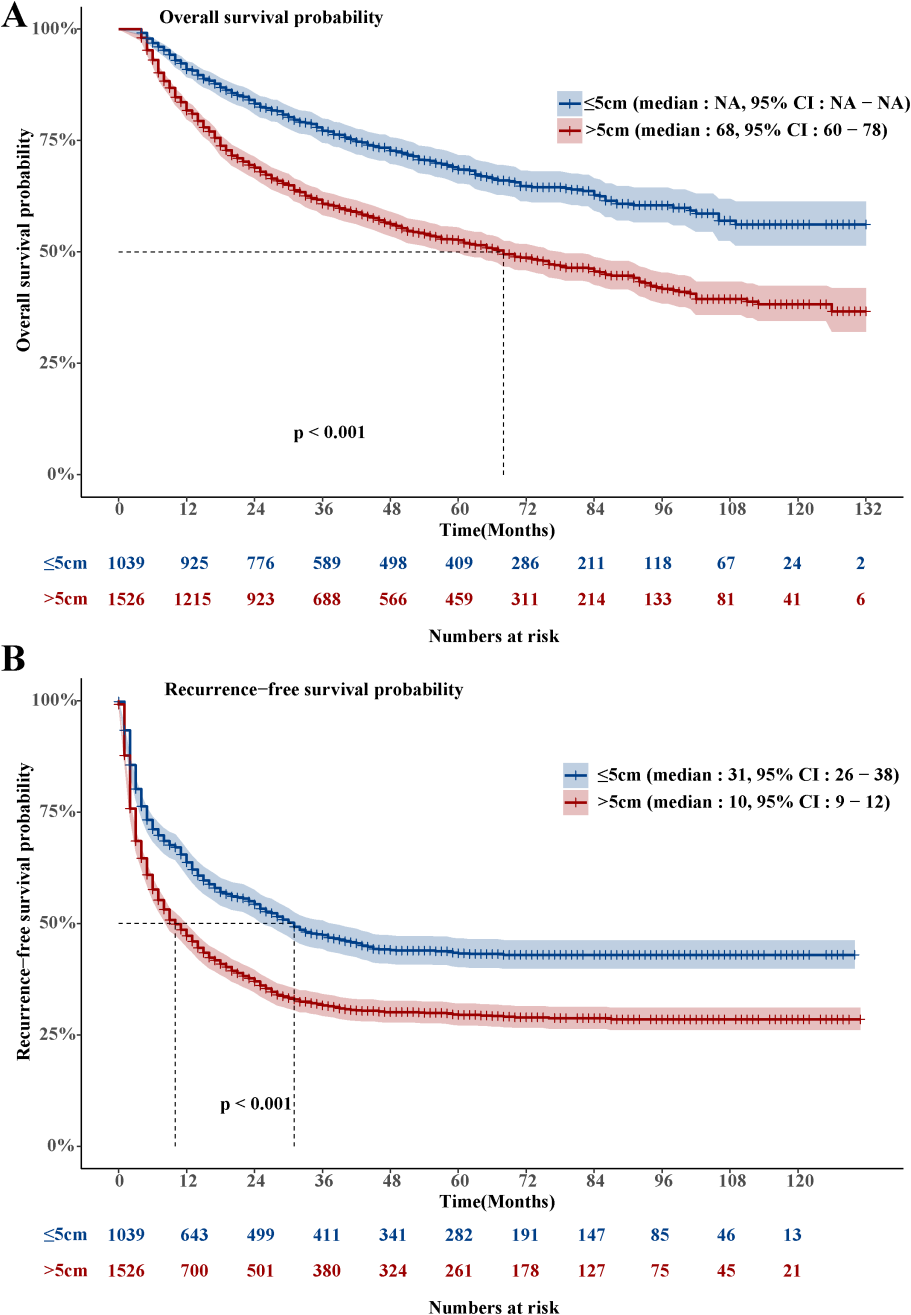
Overall survival and recurrence-free survival in the LHCC and SHCC groups.

The LHCC group’s OS was much shorter than that of the SHCC group. (Hazard Ratio, HR: 1.81, 95% Confidence Interval, CI: 1.585-2.068, **Figure 1A**). A total of 1,017 people died in the LHCC group, resulting in an overall mortality rate of 66.6%. In the LHCC group, the survival rates were 81.5% at the 1-year mark, 60.5% at the 3-year mark, and 52.2% at the 5-year mark. Similarly, the RFS of the LHCC group was significantly shorter compared to the SHCC group (HR: 1.526, 95% CI: 1.376-1.693, **Figure 1B**). A total of 1,035 people experienced recurrence in the LHCC group, leading to an overall recurrence rate of 67.8%. Among the patients in the LHCC group, the rates of RFS were 47.6% after 1 year, 32.3% after 3 years, and 30.0% after 5 years.

### 3.2 Clinicopathologic characteristics

**Figure 2:**
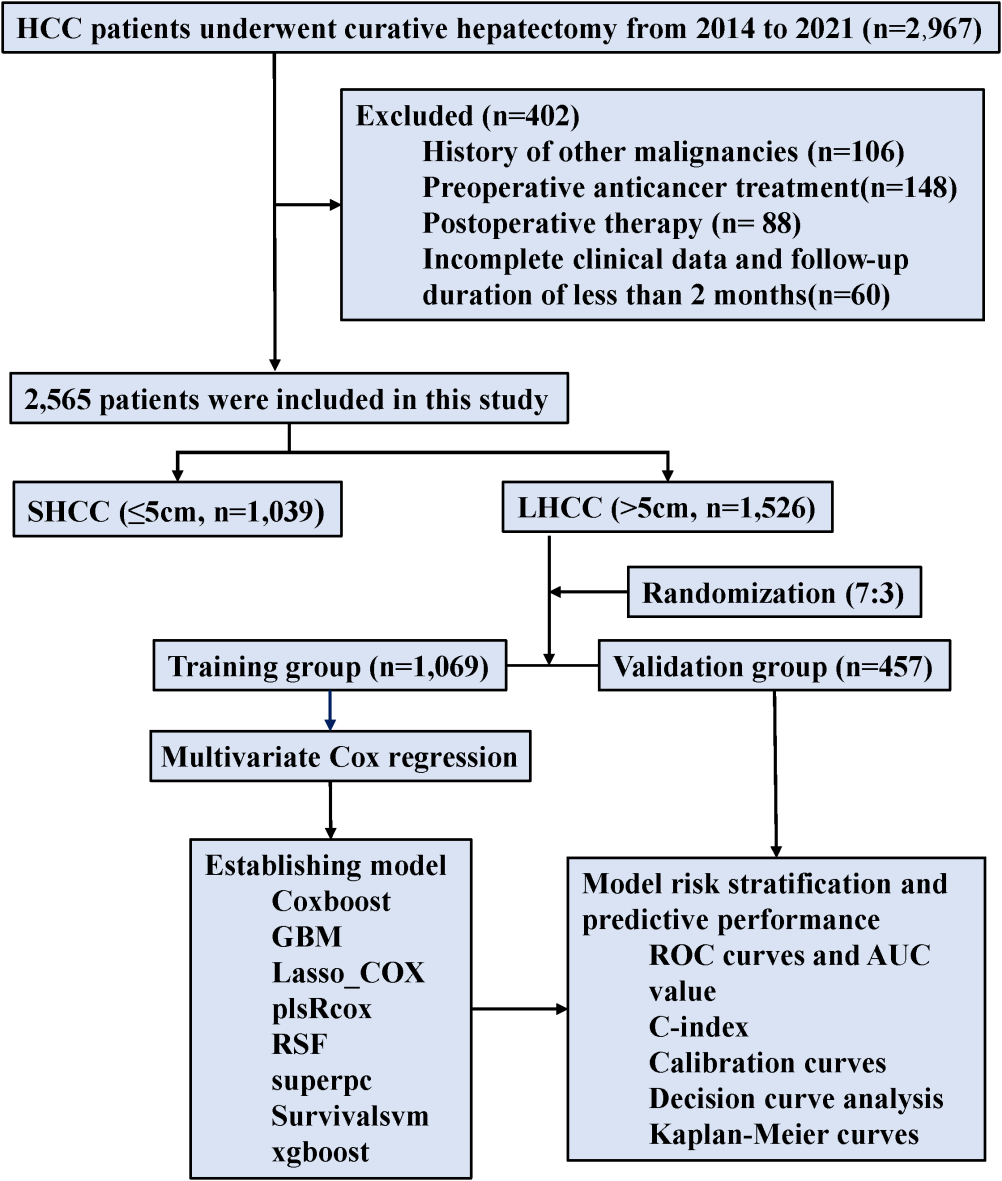
Flowchart of patient selection and analysis.

Between 2014 and 2021, 2,565 HCC patients were enrolled, including 1,526 patients with LHCC and 1,039 patients with SHCC. For additional analysis, LHCC patients were randomly split into the training group (n = 1,069) and the validation group (n = 457) in a 7:3 ratio (**Figure 2**). The baseline characteristics for both groups were presented (**Table 1**). Notably, 41.3% of patients in the training group were classified as BCLC stage B, whereas 43.5% of the validation group fell into the same category. MVI was present in 52.5% of the training group and 51.0% of the validation group. In the training group, the average tumor size was 9.20 cm, while in the validation group, it was 9.05 cm. No statistically significant differences were observed between the two groups (p>0.05). The median OS follow-up times were 42.2 months for the training and 41.5 months for the validation group.

**Table 1:** Characteristics of the training and validation groups.

Characteristic	Training (n=1,069)	Validation (n=457)	p
Gender			
Female	153 (14.3)	53 (11.6)	0.180
Male	916 (85.7)	404 (88.4)	
Age (years)			
<65	859 (80.4)	373 (81.6)	0.615
≥65	210 (19.6)	84 (18.4)	
BMI (kg/m^2^)			
<24	95 (8.9)	33 (7.2)	0.330
≥24	974 (91.1)	424 (92.8)	
Hypertension			
Absence	943 (88.2)	393 (86.0)	0.264
Presence	126 (11.8)	64 (14.0)	
Diabetes			
Absence	984 (92.0)	413 (90.4)	0.328
Presence	85 (8.0)	44 (9.6)	
Smoke			
Absence	636 (59.5)	271 (59.3)	0.989
Presence	433 (40.5)	186 (40.7)	
Family history			
Absence	886 (82.9)	372 (81.4)	0.533
Presence	183 (17.1)	85 (18.6)	
BCLC stage			
A	628 (58.7)	258 (56.5)	0.439
B	441 (41.3)	199 (43.5)	
HBsAg (ng/mL)			
Negative	168 (15.7)	85 (18.6)	0.189
Positive	901 (84.3)	372 (81.4)	
HBeAg (ng/mL)			
Negative	555 (51.9)	239 (52.3)	0.936
Positive	514 (48.1)	218 (47.7)	
HBV DNA (IU/mL)			
<500	399 (37.3)	191 (41.8)	0.113
≥500	670 (62.7)	266 (58.2)	
HCV			
Negative	1,059 (99.1)	454 (99.3)	0.811
Positive	10 (0.9)	3 (0.7)	
Number of tumors			
Single	766 (71.7)	340 (74.4)	0.300
Multiple	303 (28.3)	117 (25.6)	
Microvascular invasion			
Absence	508 (47.5)	224 (49.0)	0.632
Presence	561 (52.5)	233 (51.0)	
Child-Pugh stage			
A	989 (92.5)	425 (93.0)	0.823
B	80 (7.5)	32 (7.0)	
Total bilirubin (μmol/L)			
≤17.1	948 (88.7)	400 (87.5)	0.578
>17.1	121 (11.3)	57 (12.5)	
Albumin (g/L)			
<35	255 (23.9)	108 (23.6)	0.978
≥35	814 (76.1)	349 (76.4)	
Pre-albumin (mg/L)			
<200	683 (63.9)	283 (61.9)	0.502
≥200	386 (36.1)	174 (38.1)	
Alanine transaminase (U/L)			
<40	629 (58.8)	259 (56.7)	0.466
≥40	440 (41.2)	198 (43.3)	
Aspartate aminotransferase (U/L)			
<40	409 (38.3)	180 (39.4)	0.721
≥40	660 (61.7)	277 (60.6)	
Alpha-fetoprotein (ng/mL)			
<400	583 (54.5)	233 (51.0)	0.223
≥400	486 (45.5)	224 (49.0)	
CA19_9 (KU/L)			
≤37	943 (88.2)	404 (88.4)	0.985
>37	126 (11.8)	53 (11.6)	
Prothrombin time (s)			
<13	687 (64.3)	283 (61.9)	0.417
≥13	382 (35.7)	174 (38.1)	
Platelets (10^9^/L)			
<300	899 (84.1)	388 (84.9)	0.750
≥300	170 (15.9)	69 (15.1)	
Absolute value of white blood cell (10^9^/L)	6.76 (2.14)	6.69 (2.10)	0.594
Absolute value of lymphocyte (10^9^/L)	1.82 (1.96)	1.71 (0.60)	0.260
NLR			
<5	966 (90.4)	404 (88.4)	0.286
≥5	103 (9.6)	53 (11.6)	
Tumor size (cm)	9.20 (3.56)	9.05 (3.54)	0.461

Categorical data are n (%); Continuous data are reported as mean ±SD or as median (IQR).

BMI, body mass index; BCLC, Barcelona Clinic Liver Cancer staging system; NLR, neutrophil-lymphocyte ratio.

### 3.3 Independent risk factors associated with OS of patients with LHCC

Univariate Cox regression analysis identified significant risk factors for OS in HCC patients, including the BCLC stage (p_FDR<0.001), number of tumors (p_FDR <0.001), MVI (p_FDR <0.001), and tumor size (p_FDR <0.001). After FDR adjustment, variables including BCLC stage, number of tumors, MVI, and tumor size remained significant (P_FDR <0.05). The multivariate Cox regression analysis, which satisfied the proportional hazards assumption (Schoenfeld global test p=0.439, **Supplementary Table S3**), identified BCLC stage (HR: 1.87, 95% CI: 1.48-2.36, p<0.001), MVI (HR: 1.55, 95% CI: 1.29-1.88, p < 0.001), and tumor size (HR: 1.04, 95% CI: 1.01-1.07, p = 0.005) were associated with an independent risk factors for OS in patients with LHCC (**Supplementary Table S4**). No significant multicollinearity was detected among these variables in the multivariate model (all VIFs <5).

### 3.4 Performance of the GBM model

**Figure 3:**
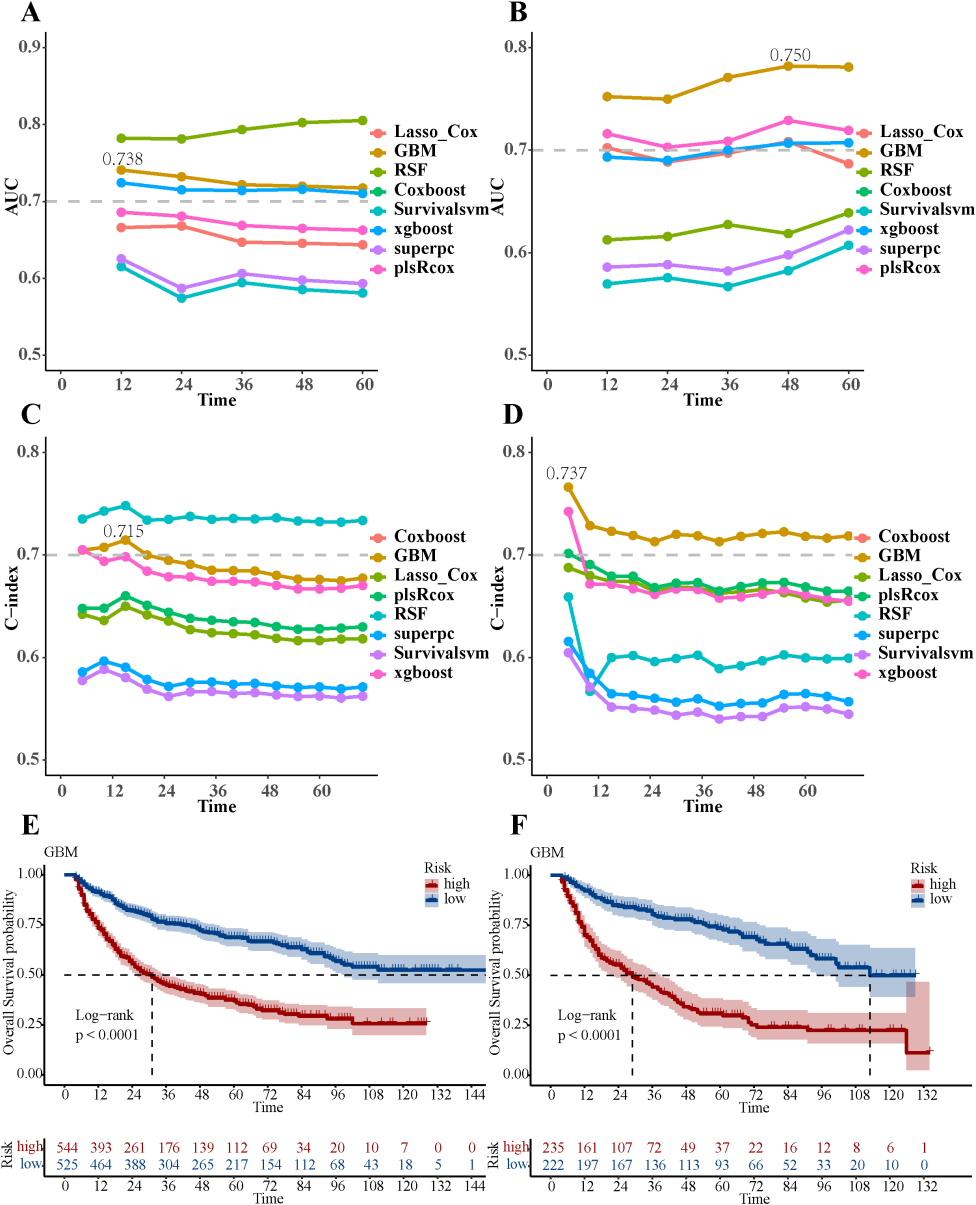
Performance evaluation of eight ML models. **(A)** The AUC values of eight ML models in the training group. **(B)** The AUC values of eight ML models in the validation group. **(C)** The C-index values of eight ML models in the training group. **(D)** The C-index values of eight ML models in the validation group. **(E)** Overall survival Kaplan-Meier curves of the GBM model in the training group. **(F)** Overall survival Kaplan-Meier curves of the GBM model in the validation group.

Among the eight ML models, the GBM model achieved AUC of 0.738 in the training group and 0.750 in the validation group, with corresponding C-index values of 0.715 and 0.737 (**Figures 3A-D**). The GBM model attained an IBS of 0.27 on the test set, indicating low overall prediction error. Compared to the null model, the GBM model demonstrated better NRI at 1-, 3-, and 5-year post-surgery, with NRI values of 32.84%, 32.74%, and 33.91%, respectively (**Supplementary Table S5**). The GBM model attained AUC values of 0.738 (95% CI: 0.696-0.780), 0.708 (95% CI: 0.675-0.740), and 0.700 (95% CI: 0.668-0.732) for 1-, 3-, and 5-year OS, respectively (**Supplementary Figures S2A-C**). The validation groups showed AUC values for 1-, 3-, and 5-year OS of 0.742 (95% CI: 0.690-0.794), 0.744 (95% CI: 0.697-0.791), and 0.750 (95% CI: 0.706-0.795), respectively (**Supplementary Figures S2D-F**). Additionally, the calibration curves of the GBM model showed improved alignment between actual observations and model predictions for 1-, 3-, and 5-year OS in both the training (**Supplementary Figure S3**) and validation groups (**Supplementary Figure S4**). The DCA curves for 1-, 3-, and 5-year OS in both the training (**Supplementary Figures S5A-C**) and validation groups (**Supplementary Figures S5D-F**) suggested potential clinical utility of the GBM model, indicating a certain degree of positive benefit under the study’s evaluation framework. Patients with low GBM scores exhibited significantly better OS outcomes than those with high GBM scores (**Figures 3E, F**). Compared to other ML models, this was evident in both the training and validation groups (**Supplementary Figures S6** and **S7**, respectively).

### 3.5 Significance of GBM features interpreted by SHAP value

**Figure 4:**
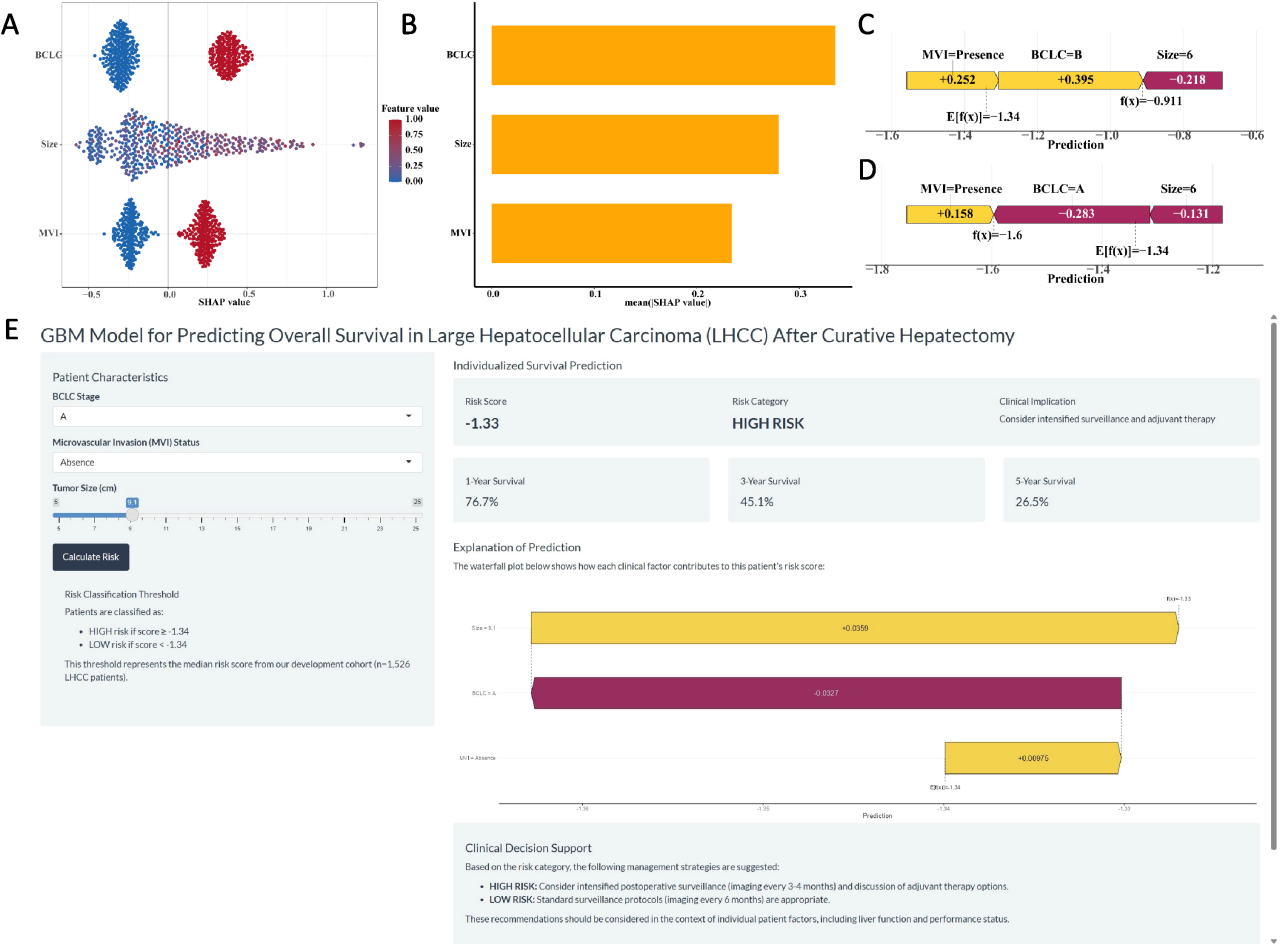
The SHAP plots of the GBM model. **(A)** The SHAP summary plot of the GBM model showed the distribution of the SHAP values of each feature. **(B)** The feature importance of GBM model variables was shown according to the mean absolute SHAP value of each feature. **(C-D)** The representative SHAP force plot of two patients with GBM risk score. **(E)** The development of an online website for clinical application of the GBM Model (https://ytx000.shinyapps.io/GBM-Shinyapp/). Clinicians input three parameters: BCLC stage, MVI status, and tumor size. The tool instantly returns an individualized prediction, including a continuous risk score and the corresponding 1-, 3-, and 5-year OS probabilities. The risk category (High or Low) is determined by comparing the calculated score to the median risk score threshold of -1.34 derived from our cohort.

The SHAP summary plot for the GBM model illustrated the influence of individual features on the predictive outcomes (**Figure 4A**). The most influential features, ranked in descending order, were BCLC stage, tumor size, and MVI (**Figure 4B**). Furthermore, SHAP force plots (**Figures 4C, D**) were applied to explain the individual predictions. For example, for patient A, with MVI, BCLC stage B, and a 6 cm tumor, the GBM model predicted a risk score of “-0.911”, so he was classified into the high-risk group. For patient B, with MVI, BCLC stage A and a 6 cm tumor, the GBM model predicted a risk score of “-1.6”, so he was classified into the low-risk group.

### 3.6 Development of web server and clinical application of GBM model

A user-friendly website (**Figure 4E**, https://ytx000.shinyapps.io/GBM-Shinyapp/) has been developed to facilitate the application of the GBM model in clinical practice. Practitioners can easily calculate individualized predicted risk scores for patients with LHCC by entering each patient’s clinical data into an online web server. Clinicians can input three key clinical parameters for LHCC patients—BCLC stage, MVI status, and tumor size—to generate individualized predictions of 1-, 3-, and 5-year OS rates. To illustrate its functionality, we present a representative case: a patient with BCLC stage A and absence of MVI, but with a large tumor size of 9.1 cm, received a GBM risk score of -1.33. As this score is higher than the mean risk score threshold of -1.34 used in our study, the model classified this patient into the high-risk group. The corresponding predicted 1-, 3-, and 5-year OS rates for this individual were 76.70%, 45.12%, and 26.54%, respectively. This example underscores the model’s ability to identify high-risk patients even among those with otherwise favorable clinical features (early BCLC stage and no MVI), highlighting the critical prognostic weight of tumor size captured by the GBM algorithm.

### 3.7 Comparison of GBM model with previous postoperative predictive models

**Figure 5:**
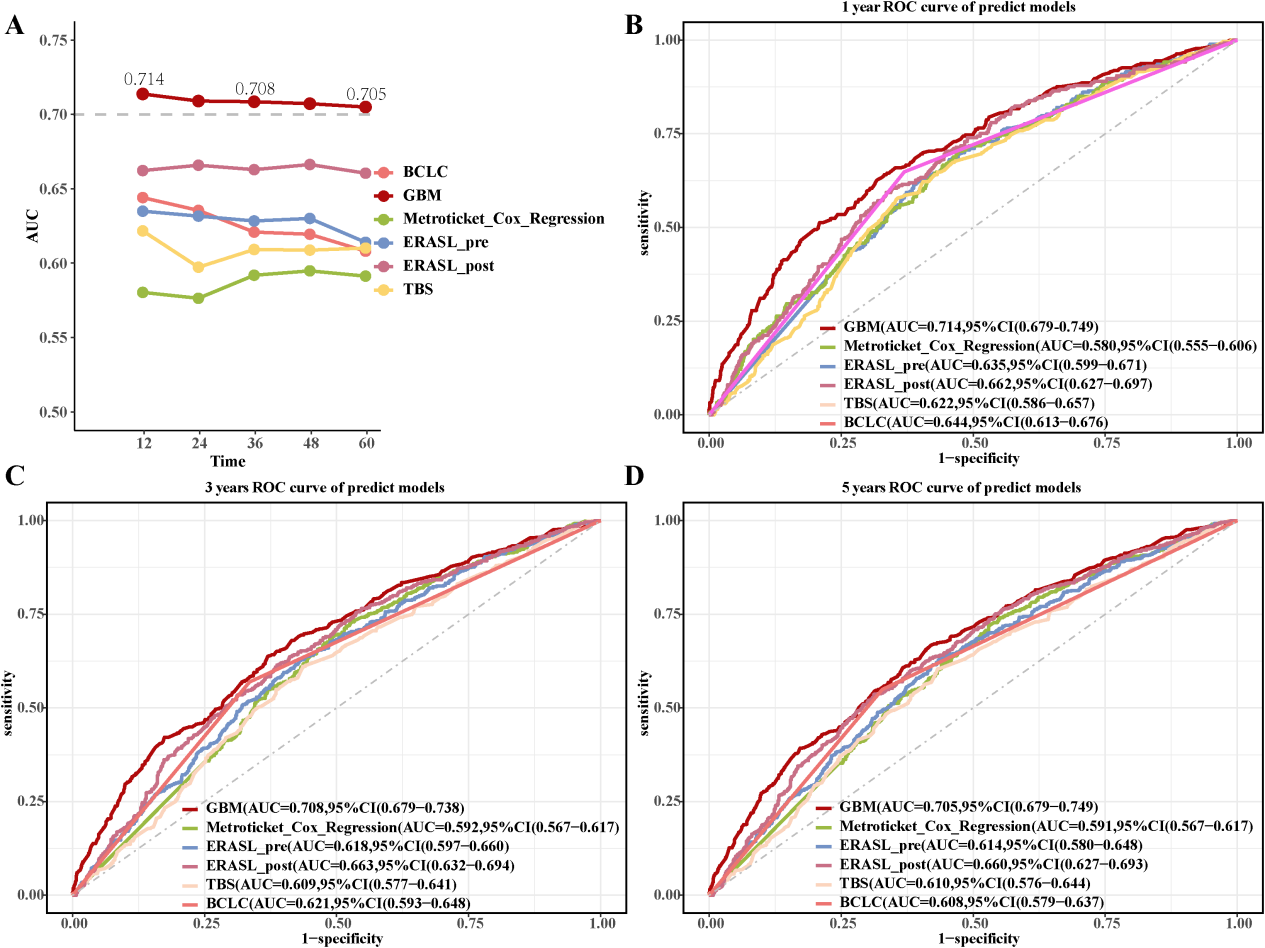
Comparison of the GBM model with previous postoperative predictive models. **(A)** The AUC values of the GBM model with previous postoperative predictive models. **(B-D)** The 1-year, 3-year, and 5-year ROC curves of the GBM model and previous postoperative predictive models.

We compared the performance of the GBM model with previous postoperative predictive models (**Supplementary Table S6**). The GBM model achieved AUC values of 0.714 (95% CI: 0.679-0.749), 0.708 (95% CI: 0.679-0.738), and 0.705 (95% CI: 0.674-0.736) for 1-, 3-, and 5-year OS, respectively (**Figures 5A-D**). The GBM model reached C-index values of 0.680, 0.663, and 0.656 that correspond to 1-, 3-, and 5-year OS, respectively (**Supplementary Figure S8A**). The DCA curves (**Supplementary Figures S8B-D**) for 1-, 3-, and 5-year OS showed the GBM model’s strong clinical utility and superior net benefit. The calibration curves (**Supplementary Figure S9**) for the GBM model showed improved alignment between predicted probabilities and observed outcomes for 1-, 3-, and 5-year OS.

### 3.8 Differential performance of the GBM model stratified by neutrophil-lymphocyte ratio

**Figure 6:**
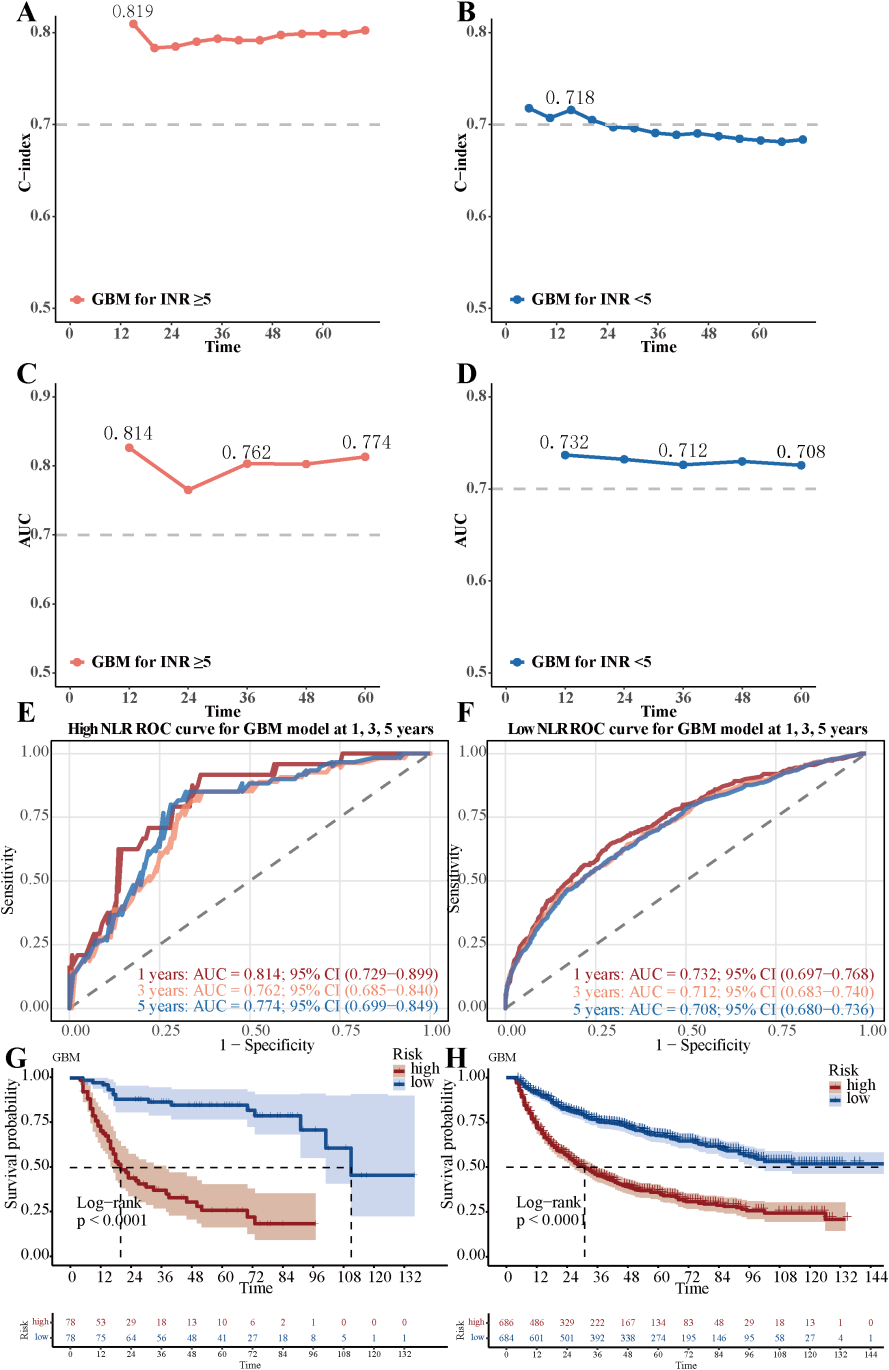
Differential performance of the GBM model stratified by Neutrophil-lymphocyte ratio. **(A)** The C-index value of GBM model in the low neutrophil-lymphocyte ratio group. **(B)** The C-index value of GBM model in the high neutrophil-lymphocyte ratio group. **(C)** The AUC value of GBM model in the low neutrophil-lymphocyte ratio group. **(D)** The AUC value of GBM model in the high neutrophil-lymphocyte ratio group. **(E)** The 1-year, 3-year, and 5-year ROC curves of the GBM model in the low neutrophil-lymphocyte ratio group. **(F)** The 1-year, 3-year, and 5-year ROC curves of the GBM model in the high neutrophil-lymphocyte ratio group. **(G)** Overall survival Kaplan-Meier curves of the GBM model in the low neutrophil-lymphocyte ratio group (NLR <5, n=1,370), stratified by the GBM model risk score (high vs. low). **(H)** Overall survival Kaplan-Meier curves of the GBM model in the high neutrophil-lymphocyte ratio group (NLR ≥5, n=156), stratified by the GBM model risk score. The difference between the survival curves was assessed by the log-rank test (p < 0.01 for both comparisons).

Notably, the GBM model demonstrated differential predictive performance between patients with high (NLR ≥5, n=156) and low (NLR <5, n=1,370) neutrophil-lymphocyte ratios, using a cutoff value supported by prior literature (26–28). The GBM model achieved a C-index of 0.819 in the high NLR group and 0.718 in the low NLR group, with corresponding AUC values of 0.814 and 0.732 (**Figures 6A-D**). The GBM model attained AUC values of 0.814 (95% CI: 0.729-0.899), 0.762 (95% CI: 0.685-0.840), and 0.774 (95% CI: 0.699-0.849) for 1-, 3-, and 5-year OS in high NLR groups, respectively (**Figure 6E**).The low NLR groups showed AUC values for 1-, 3-, and 5-year OS of 0.732 (95% CI: 0.697-0.768), 0.712 (95% CI: 0.683-0.740), and 0.708 (95% CI: 0.680-0.736), respectively (**Figure 6F**). Patients with low GBM scores exhibited significantly better OS outcomes than those with high GBM scores in both the high NLR group (log-rank p <0.01; **Figure 6G**) and the low NLR group (log-rank p <0.01; **Figure 6H**).

## 4 Discussion

Compared with SHCC patients, those with LHCC had a significantly reduced OS and RFS. This discrepancy was likely attributed to tumor size. Larger tumors frequently involve multiple hepatic segments and are often located near major vascular structures. Moreover, larger tumors are more likely to be associated with MVI or satellite nodules. These factors culminate in a high level of tumor heterogeneity in LHCC. Moreover, they intricately complicate the processes of hepatectomy (29–31). Conversely, smaller tumors are typically confined to a single hepatic segment or remain within the boundaries of the same hepatic lobe, even when multiple tumors are present. In such situations, hepatectomy usually yields favorable outcomes (32). Beyond its established role as a histological marker of invasiveness, MVI may signify a profoundly permissive tumor immune microenvironment (TIME) that is instrumental in facilitating immune escape (33, 34). This permissive TIME is characterized by a loss of cytotoxic effector cells, such as CD8+ T cells and B lymphocytes, and a relative increase in immunosuppressive populations (35). The process of intravascular infiltration is mechanistically linked to programs like epithelial-mesenchymal transition (EMT). Importantly, such adaptations not only enhance cellular motility but are also increasingly recognized to directly promote immune evasion. This can occur through mechanisms that impair tumor antigen presentation and, critically, through the upregulation of immunosuppressive checkpoints like PD-L1 (36, 37). Consequently, the potent predictive power of MVI in our model likely reflects this underlying immunosuppressive phenotype, a hallmark of aggressive cancers that enables tumors to evade host immunity and ultimately drive both local recurrence and distant metastasis (33, 38).

This study developed a novel ML model utilizing the GBM algorithm to predict the prognosis of LHCC based on data from 1,526 patients with LHCC who underwent curative hepatectomy. Among the eight ML models evaluated in this study, the GBM model showed relatively better information fitting capacity and preliminarily captured the complex relationships between risk factors and patient survival, demonstrating promising predictive performance in our group. Notably, this novel model outperformed existing postoperative prediction models. We utilized SHAP to thoroughly investigate the influence of features on the GBM model’s decision-making process.

The GBM model demonstrated superior predictive performance compared to seven other ML algorithms. Its excellence can be attributed to three key mechanisms: Firstly, GBM’s iterative optimization of residuals enabled it to effectively capture subtle signals, even in studies with limited patient samples. This is essential for HCC research. Secondly, the model’s regularization parameters (39, 40), including a shrinkage value of 0.01 and tree depth control (interaction depth=5), ensured a balance between overfitting and model complexity, preventing the model from being overly adapted to the training information. Finally, the use of the Cox partial likelihood as the survival loss function improved the consistency in survival time ranking, enhancing the clinical relevance of predictions. These findings are consistent with previous studies that suggest GBM may have certain advantages in similar scenarios (36, 41, 42).

In addition, we have demonstrated how the individualized SHAP waterfall chart (43) can transform abstract risk scores into specific and actionable decision nodes. These nodes were designed to be accessible and comprehensible within a clinical context, while effectively reflecting the model’s output. Unlike previous postoperative prediction models, we have unraveled the “black box” decision-making process inherent to the GBM model, enabling clinicians to confidently rely on its predictive outcomes. Through feature importance ranking, the BCLC staging system emerged as the most critical factor. Comparatively, BCLC incorporated several tumor-related characteristics, such as the number of tumors, extrahepatic metastasis, and vascular invasion status (44). Moreover, BCLC integrated crucial factors, including the Child-Pugh score for liver function and the ECOG PS score, which reflects the patient’s overall condition. These aspects serve as valuable reference points for predicting the OS prognosis of LHCC patients after curative hepatectomy^25.^ However, prior studies have indicated that the BCLC staging system has limitations in accurately predicting OS, signaling the need for improved predictive methodologies (45, 46).

In this study, MVI was again identified as an important prognostic feature in our GBM model, which is consistent with findings in many cancer types where vascular invasion is associated with immunosuppressive microenvironments and poor clinical outcomes (33, 38). Previous studies indicated that MVI represented a critical process through which aggressive tumor cells remodel the TME to foster immune escape, primarily through recruitment of immunosuppressive cells via cytokine signaling (34, 47, 48). Specifically, MVI-positive tumors secreted cytokines (e.g., VEGF, TGF-β, IL-10) that recruit immunosuppressive cells including myeloid-derived suppressor cells and regulatory T cells, creating an “immune desert” characterized by diminished cytotoxic T-cell infiltration and function (33, 36). This immunosuppressive landscape was further compounded by the frequent upregulation of PD-L1 in MVI-capable cells, which directly inhibits T-cell function through PD-1/PD-L1 checkpoint interaction, thereby facilitating immune evasion and early recurrence (36, 37). Previous studies have identified MVI as a major risk factor contributing to high recurrence risk in patients, urging clinicians to consider MVI status when making clinical decisions and formulating treatment plans (49). Early MVI was defined as small clusters of malignant cells located at the margin of the primary lesion, while late MVI referred to the presence of scattered malignant cell clusters across the liver. The formation of MVI may be linked to the activation of the epithelial-mesenchymal transition transcriptional program, which could explain its association with poor post-hepatectomy prognosis in patients with LHCC (50, 51). Furthermore, tumor size was the second most significant feature in the GBM model. Unlike the BCLC staging system, which categorized tumor size based on designated specific cut-off values, this analysis treated tumor size as a continuous variable (52, 53). This approach provided a more nuanced reflection of the TBS for patients with LHCC. The TBS had proven to be a vital parameter in assessing the OS prognosis of patients with HCC (54, 55).

Compared with previous postoperative predictive models (56, 57), the GBM model was specifically focused on predicting the prognosis of LHCC patients after curative hepatectomy. Although Zhong’s ERASL-pre/post model (57) incorporated the MVI indicator, it still prioritized early recurrence prediction. The GBM model integrated the postoperative pathological feature of MVI, enhancing its ability to predict the long-term OS and overcome the ERASL models’ narrow focus on short-term recurrence outcomes. In addition, compared with Zeng et al.’s nomograms for predicting outcomes in patients with LHCC after curative hepatectomy (58), which were constructed using cox regression, the GBM algorithm offered distinct advantages in handling nonlinear relationships. What’s more, the GBM model employed the SHAP to transparently visualize how variables like BCLC stage, tumor size, and MVI influence predictions. Under the interpretability evaluation criteria adopted in this study, the GBM model’s interpretability performance was better than that of traditional multivariable analyses, which may help clinicians better understand the model’s decision-making logic within the framework of this study and enhance their confidence in its potential clinical application. Unlike the shiny online tool of the nomogram, which was limited to fixed-formula calculations, the GBM model leveraged ML algorithms to generate dynamic predictions (59), which may provide the possibility of real-time adaptation to patient-specific data changes under appropriate conditions.

Our study found that the GBM model demonstrated better performance in patients with high NLR compared to those with low NLR group (C-index: 0.819 vs. 0.718). This finding may be explained by immunological mechanisms. The elevated NLR reflected a systemic inflammatory state where neutrophils promote immunosuppression through the release of neutrophil extracellular traps (NETs) and suppression of CD8+ T cell function (60, 61), while lymphocytopenia directly impaired anti-tumor immune responses (62). Previous studies have indicated that the poor prognosis of HCC results from the combined effects of MVI and high immunosuppressive state (60, 63). In our study, the GBM model effectively captured this synergistic effect, which may provide a reference for clinical stratification and intervention.

While our SHAP analysis effectively underscored the significance of established clinical risk factors such as BCLC stage, MVI, and tumor size, we recognized that the inclusion of novel biomarkers and complex feature interactions might further refine the model’s predictive capability. Future studies could explore the integration of multi-omics data—such as genomic (e.g., TP53 or CTNNB1 mutations), transcriptomic (e.g., immune signatures, EMT profiles), or radiomic features—which may not only enhance prognostic accuracy but also contribute to a more comprehensive mechanistic understanding of tumor heterogeneity, immune evasion, and the aggressive behavior of LHCC. These insights could potentially offer new directions for therapeutic targeting.

The present study has several limitations. Firstly, the primary limitation of the current study is its single-center design with relatively small sample size. Secondly, the lack of external cohort also requires further validation of the reliability for our GBM model. The patient population, surgical techniques, and perioperative management protocols are all specific to our institution. This homogeneity limits the generalizability of our GBM model to other centers with different patient demographics and clinical practices. Third, although we collected a broad set of clinical and laboratory variables, our feature selection was necessarily stringent to avoid overfitting, potentially omitting more complex or novel biomarkers. More importantly, the model does not incorporate advanced omics data (e.g., genomic, transcriptomic, or radiomic features), which could significantly enhance predictive performance and biological interpretability. Fourth, our model is a static prediction model based solely on preoperative parameters. Thus, multi-center and large-scale randomized controlled trials are necessary to confirm the clinical application value of our GBM model. The development of a dynamic prediction model that updates risk estimates over time based on new clinical data represents a valuable and necessary future direction to further enhance clinical utility.

To sum up, we have developed and internally validated a novel prognostic prediction model utilizing the GBM ML algorithm. The model demonstrated promising performance in stratifying the survival risk of LHCC patients within our group. While these results are positive, external validation in independent, multi-center populations is imperative to confirm its generalizability and ultimate clinical utility.

## Data Availability

The raw data supporting the conclusions of this article will be made available by the authors, without undue reservation.

## Supplementary material

Supplementary Figure 1 Lasso-Cox regression for identifying prognostic factors in LHCC patients after curative hepatectomy. **(A)** Partial likelihood deviance plot as a function of log-transformed penalty parameter lambda value. **(B)** Coefficient profile plot from LASSO regression.

Supplementary Figure 2 The 1-, 3-, and 5-year ROC curves of eight ML prognosis models. **(A-C)** The 1-, 3-, and 5-year ROC curves of eight ML models in the training groups. **(D-F)** The 1-, 3-, and 5-year ROC curves of eight ML models in the validation groups.

Supplementary Figure 3 The 1-, 3-, and 5-year calibration curves of eight ML models in the training group.

Supplementary Figure 4 The 1-, 3-, and 5-year calibration curves of eight ML models in the validation group.

Supplementary Figure 5 The 1-, 3-, and 5-year DCA curves of eight ML models. **(A, B)** The 1-, 3-, and 5-year DCA curves of eight ML models in the training groups. **(C, D)** The 1-, 3-, and 5-year DCA curves of eight ML models in the validation groups.

Supplementary Figure 6 The Kaplan-Meier curves of eight ML models in the training group.

Supplementary Figure 7 The Kaplan-Meier curves of eight ML models in the validation group.

Supplementary Figure 8 Comparison of the predictive performance of the GBM model and previous prognostic models. **(A)** The C-index values of the GBM model and previous postoperative predictive models. **(B-D)** The 1-year, 3-year, and 5-year DCA curves of the GBM model and previous postoperative predictive models.

Supplementary Figure 9 The 1-, 3-, and 5-year calibration curves of the GBM model and previous postoperative predictive models.

Supplementary Table 1 The parameters of the machine learning algorithms.

Supplementary Table 2 Discriminative performance of eight ML models evaluated by C-index in stratified 10-Fold cross-validation and independent internal validation.

Supplementary Table 3 The Schoenfeld residual tests of the multivariate Cox regression.

Supplementary Table 4 Univariable and multivariable Cox regression analysis of factors in the training group.

Supplementary Table 5 IBS in training and test sets and NRI at 1-, 3-, and 5-year follow-up for different ML models.

Supplementary Table 6 Predictive performance of the previous postoperative predictive models.
