# Supplementary figures and images for: Development of a machine learning model to predict overall survival for large hepatocellular carcinoma at BCLC stage A or B after curative hepatectomy
Source: Front Immunol. 2025 Oct 21;16:1640075. doi: 10.3389/fimmu.2025.1640075 (PMC12583128; doi:10.3389/fimmu.2025.1640075)

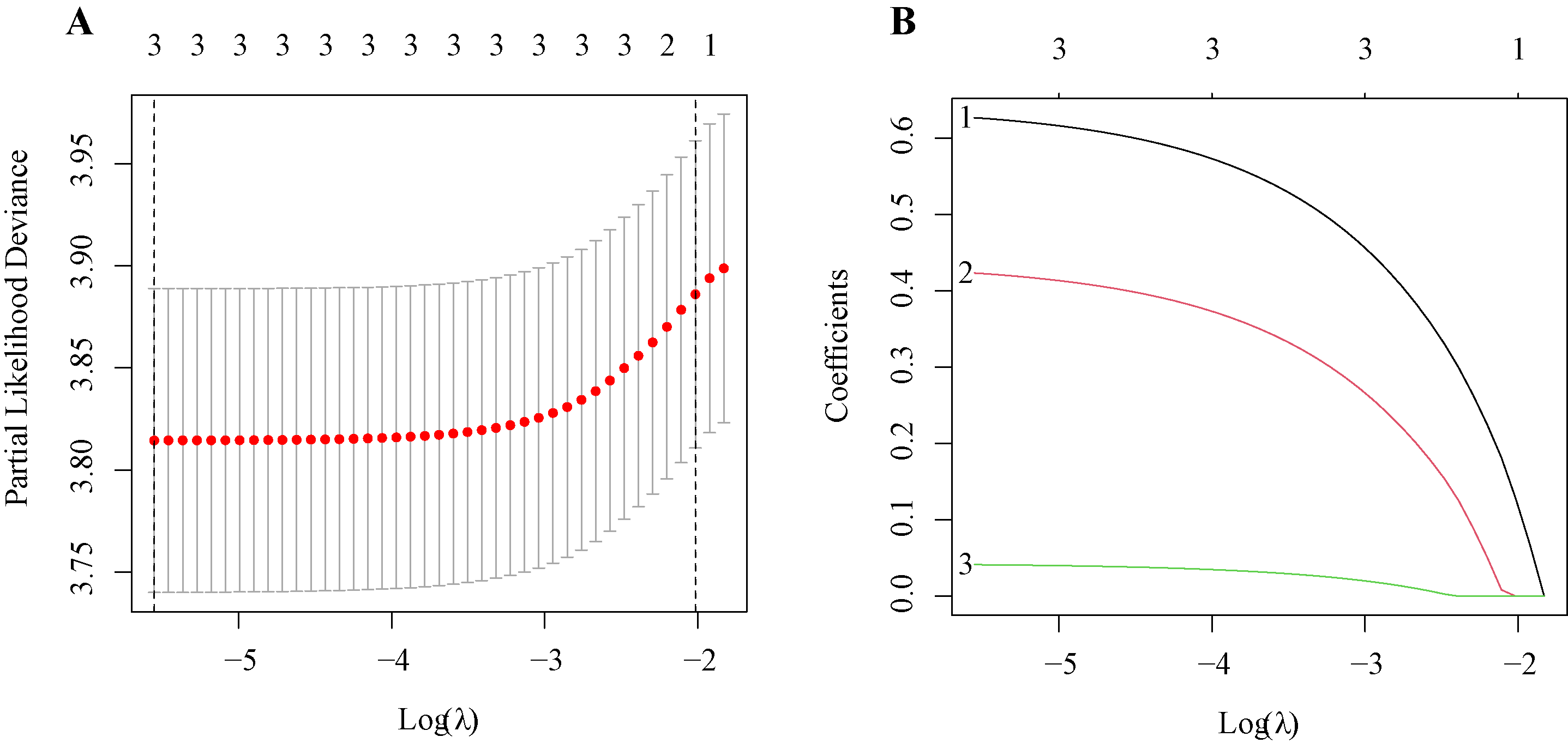

Supplement: Supplementary Figure 1 — Lasso-Cox regression for identifying prognostic factors in LHCC patients after curative hepatectomy. (A) Partial likelihood deviance plot as a function of log-transformed penalty parameter lambda value. (B) Coefficient profile plot from LASSO regression. [file Image1.tif]

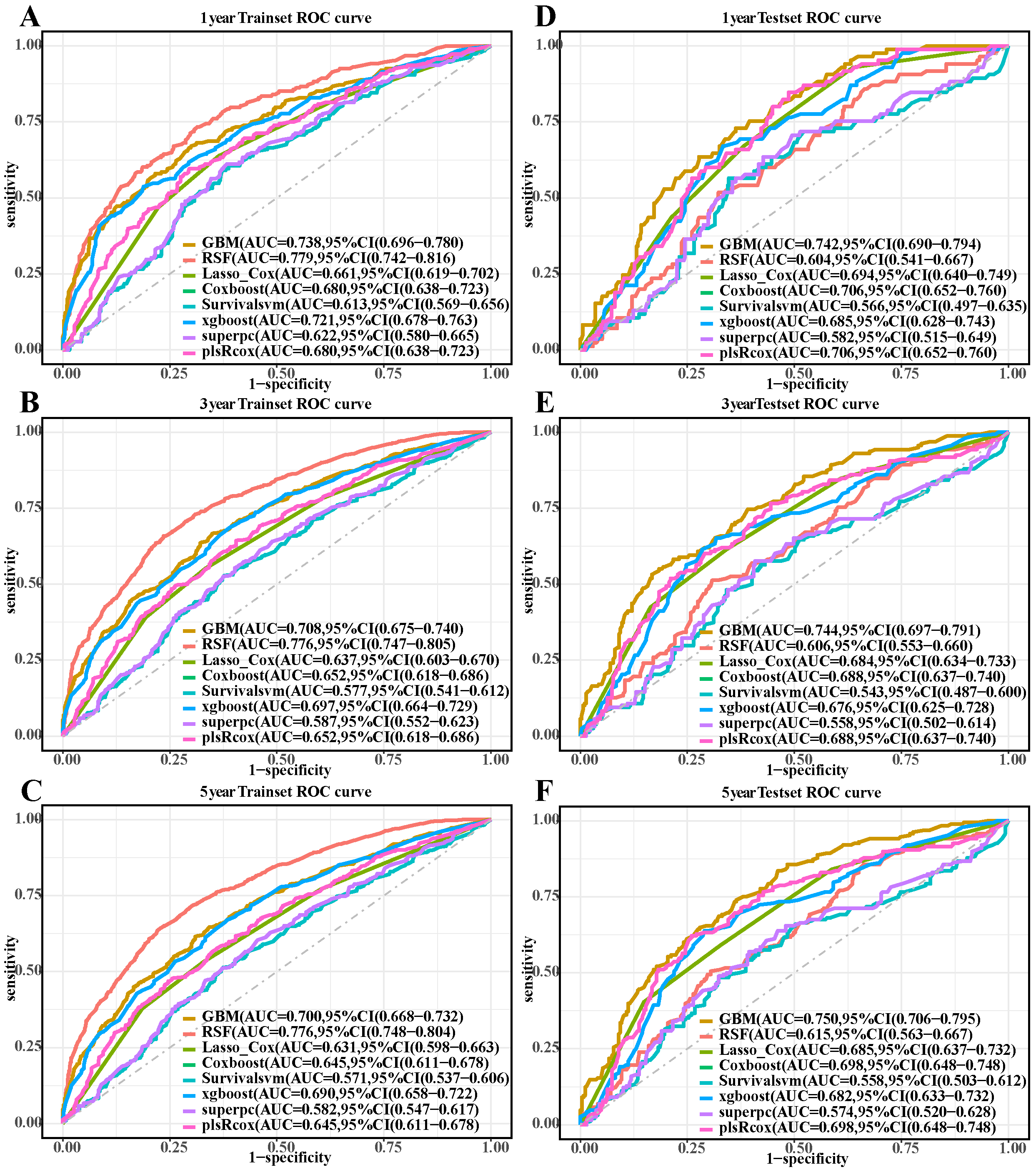

Supplement: Supplementary Figure 2 — The 1-, 3-, and 5-year ROC curves of eight ML prognosis models. (A-C) The 1-, 3-, and 5-year ROC curves of eight ML models in the training groups. (D-F) The 1-, 3-, and 5-year ROC curves of eight ML models in the validation groups. [file Image2.tif]

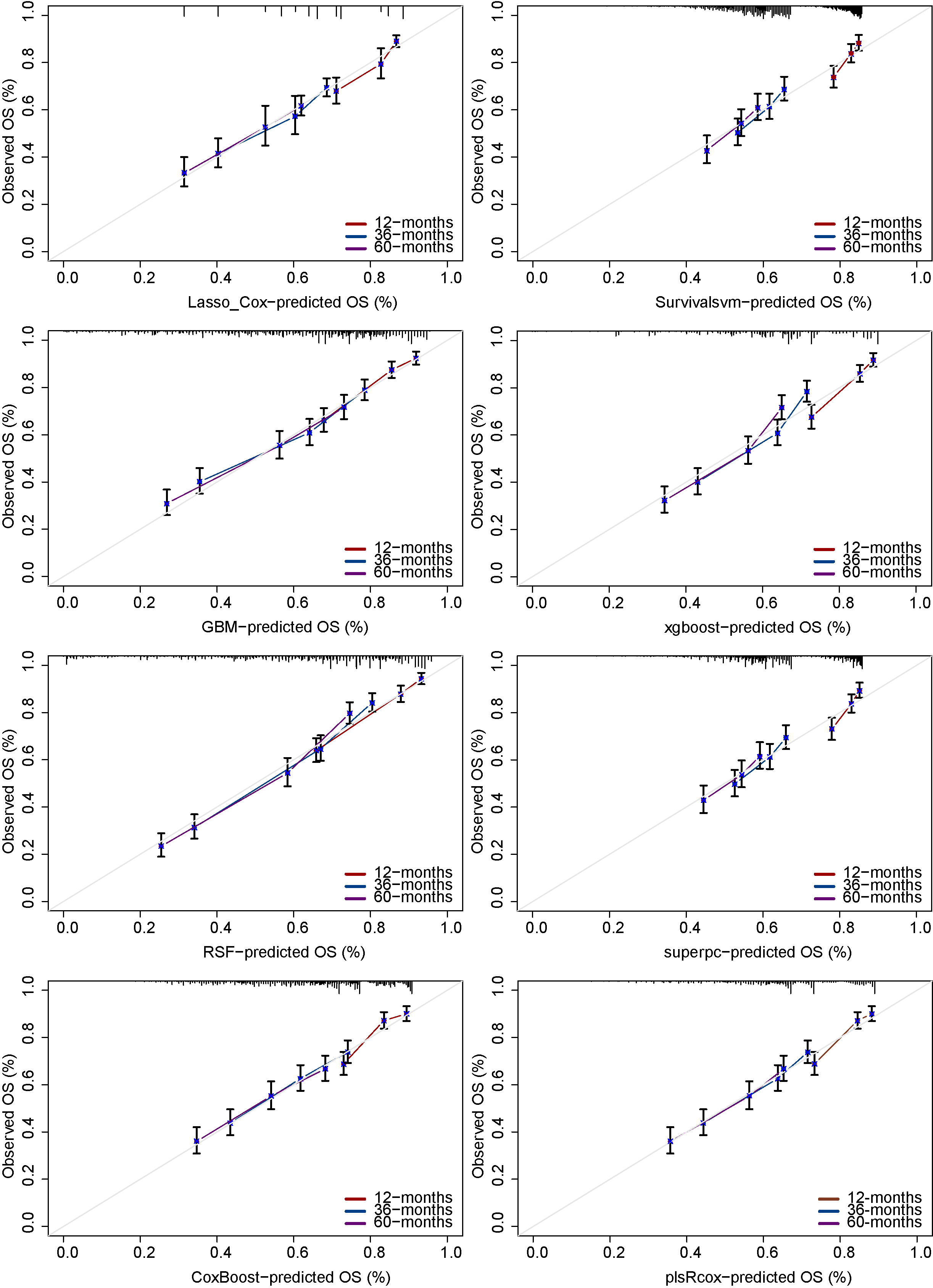

Supplement: Supplementary Figure 3 — The 1-, 3-, and 5-year calibration curves of eight ML models in the training group. [file Image3.tif]

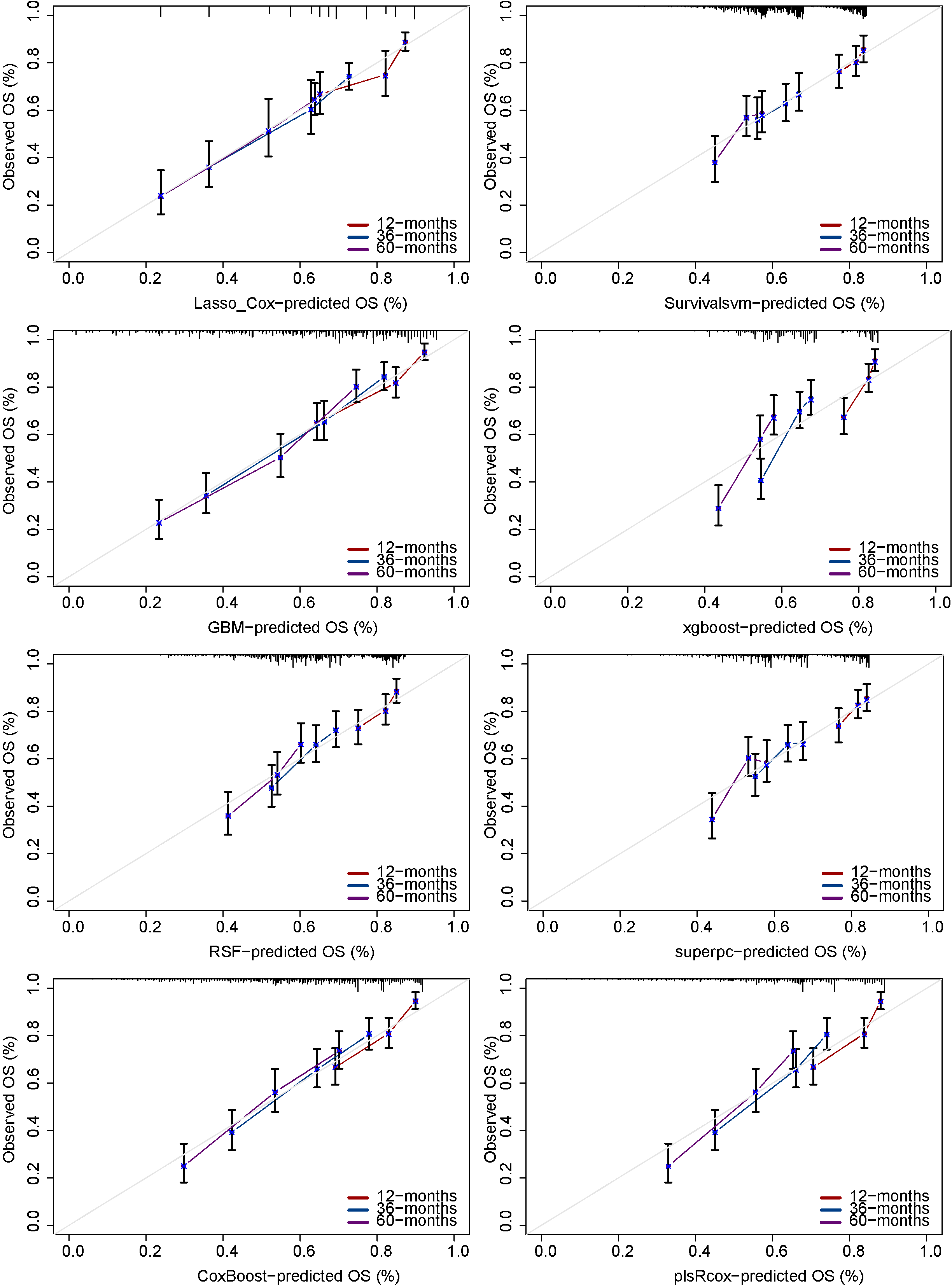

Supplement: Supplementary Figure 4 — The 1-, 3-, and 5-year calibration curves of eight ML models in the validation group. [file Image4.tif]

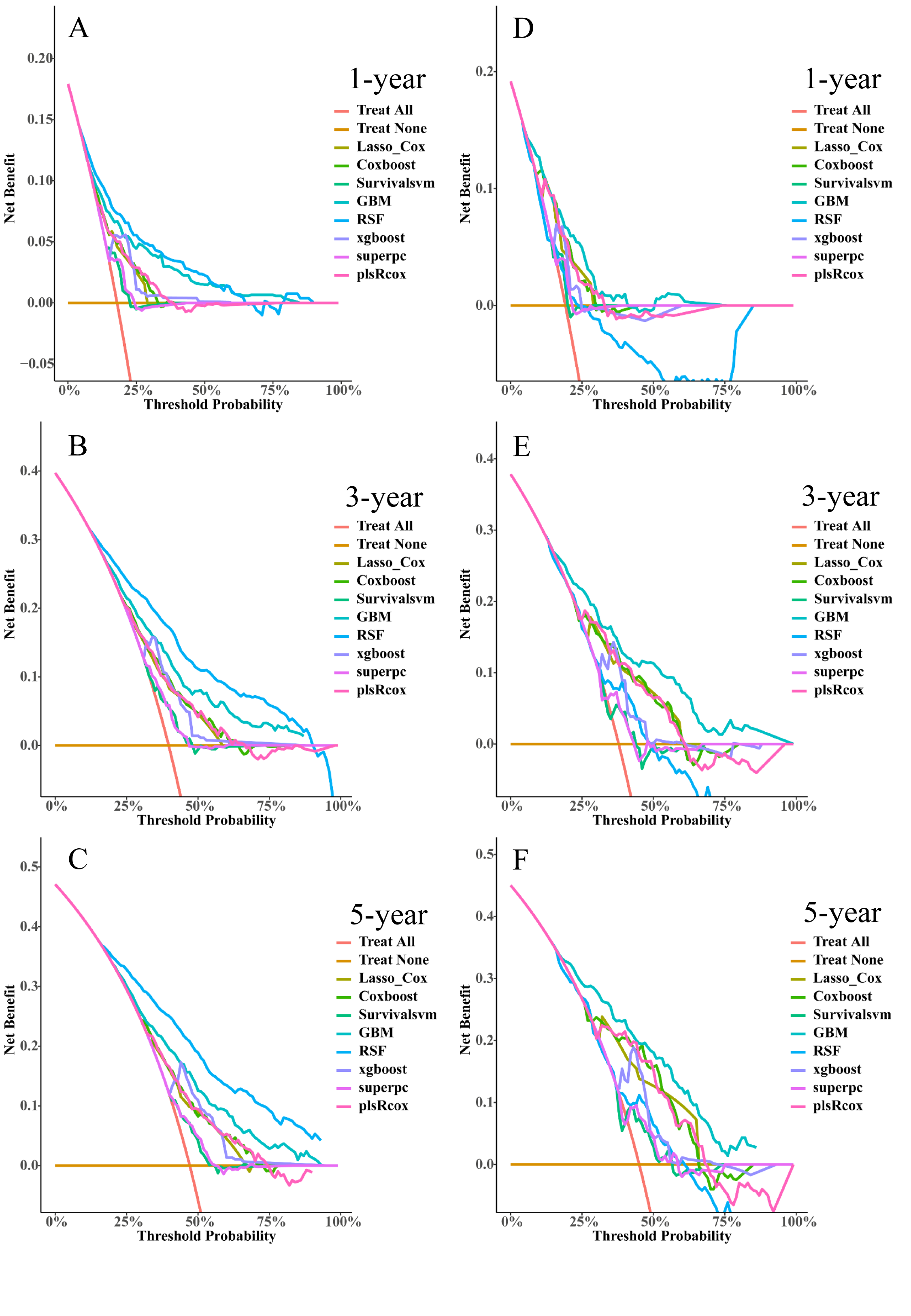

Supplement: Supplementary Figure 5 — The 1-, 3-, and 5-year DCA curves of eight ML models. (A, B) The 1-, 3-, and 5-year DCA curves of eight ML models in the training groups. (C, D) The 1-, 3-, and 5-year DCA curves of eight ML models in the validation groups. [file Image5.tif]

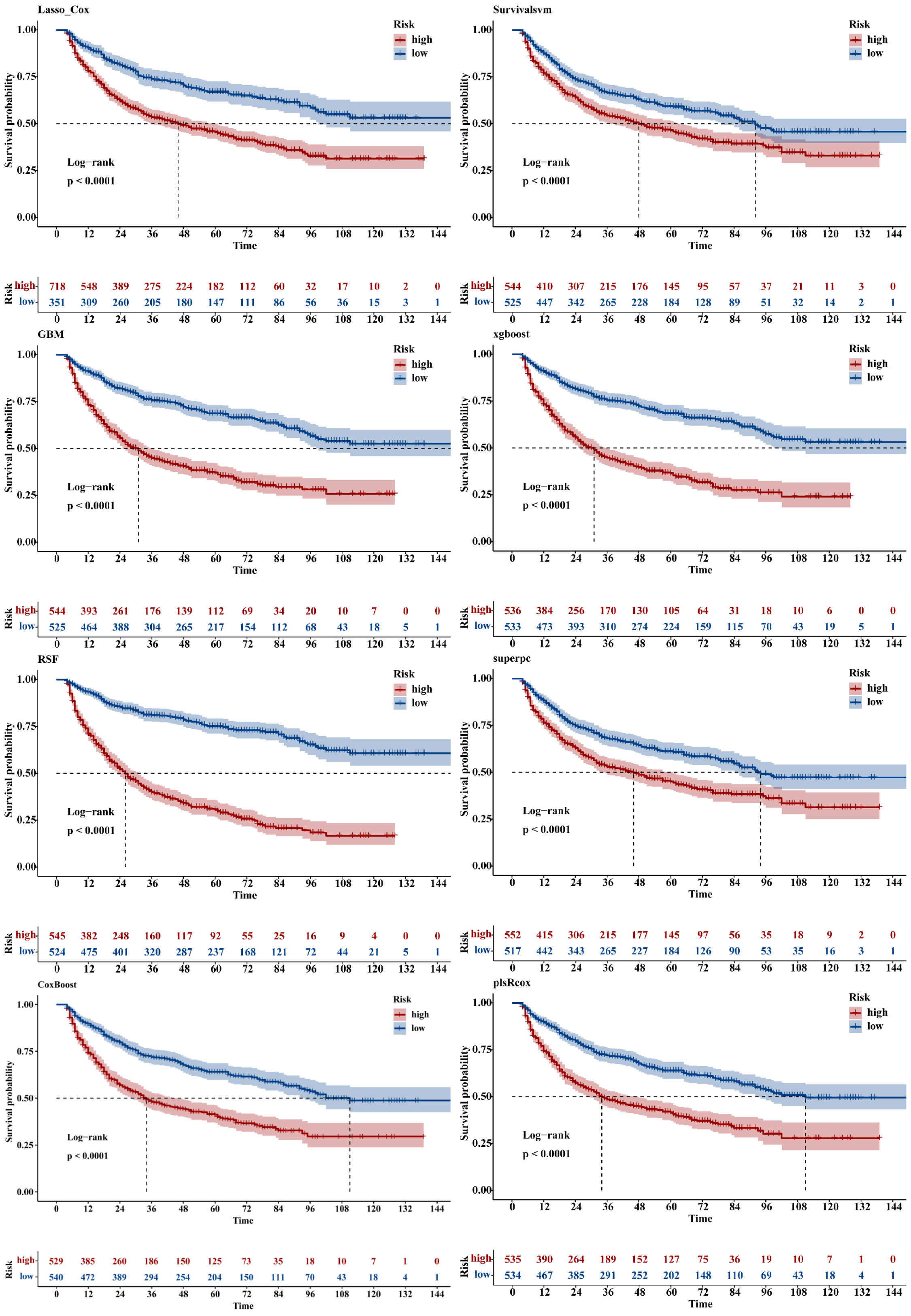

Supplement: Supplementary Figure 6 — The Kaplan-Meier curves of eight ML models in the training group. [file Image6.tif]

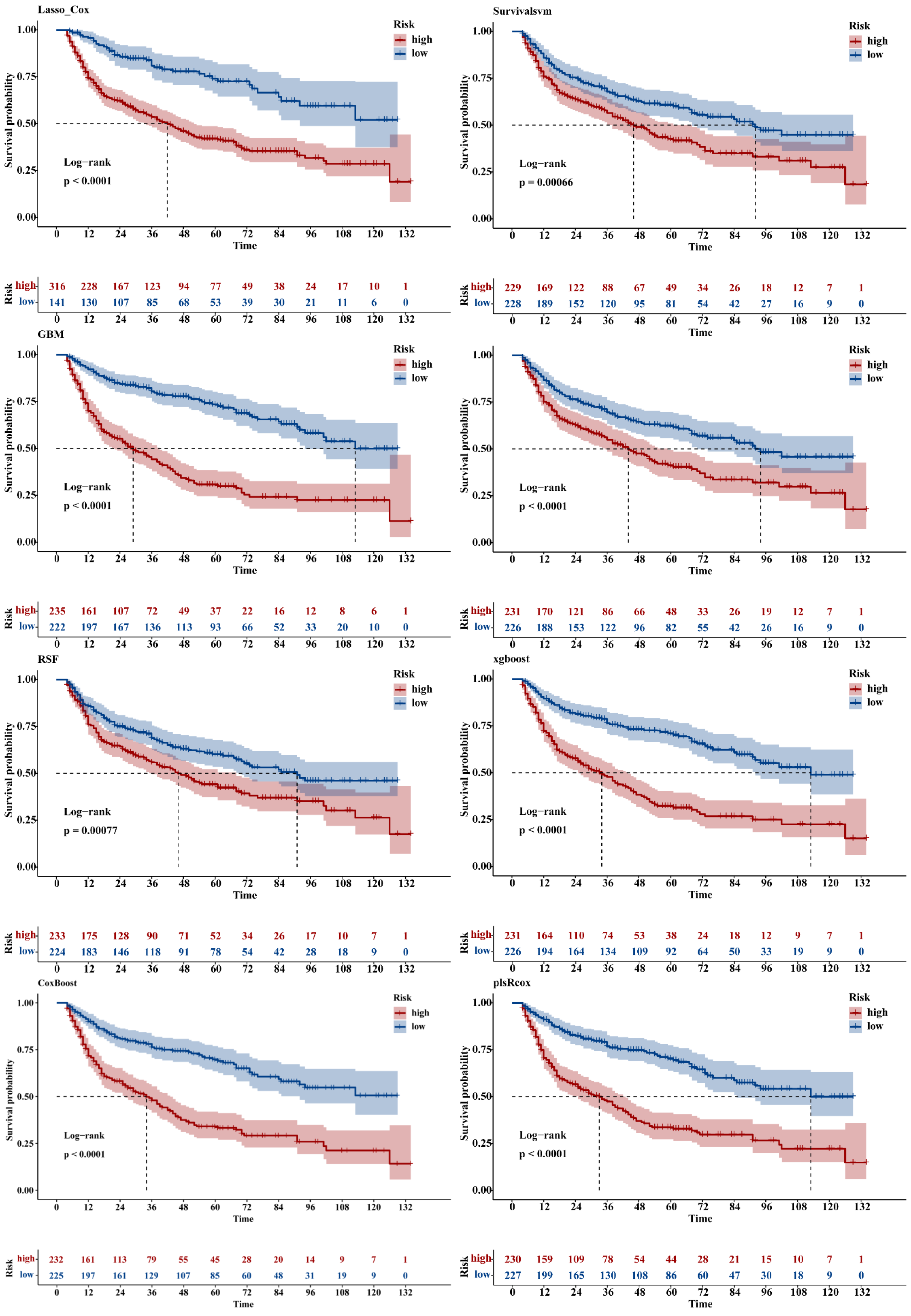

Supplement: Supplementary Figure 7 — The Kaplan-Meier curves of eight ML models in the validation group. [file Image7.tif]

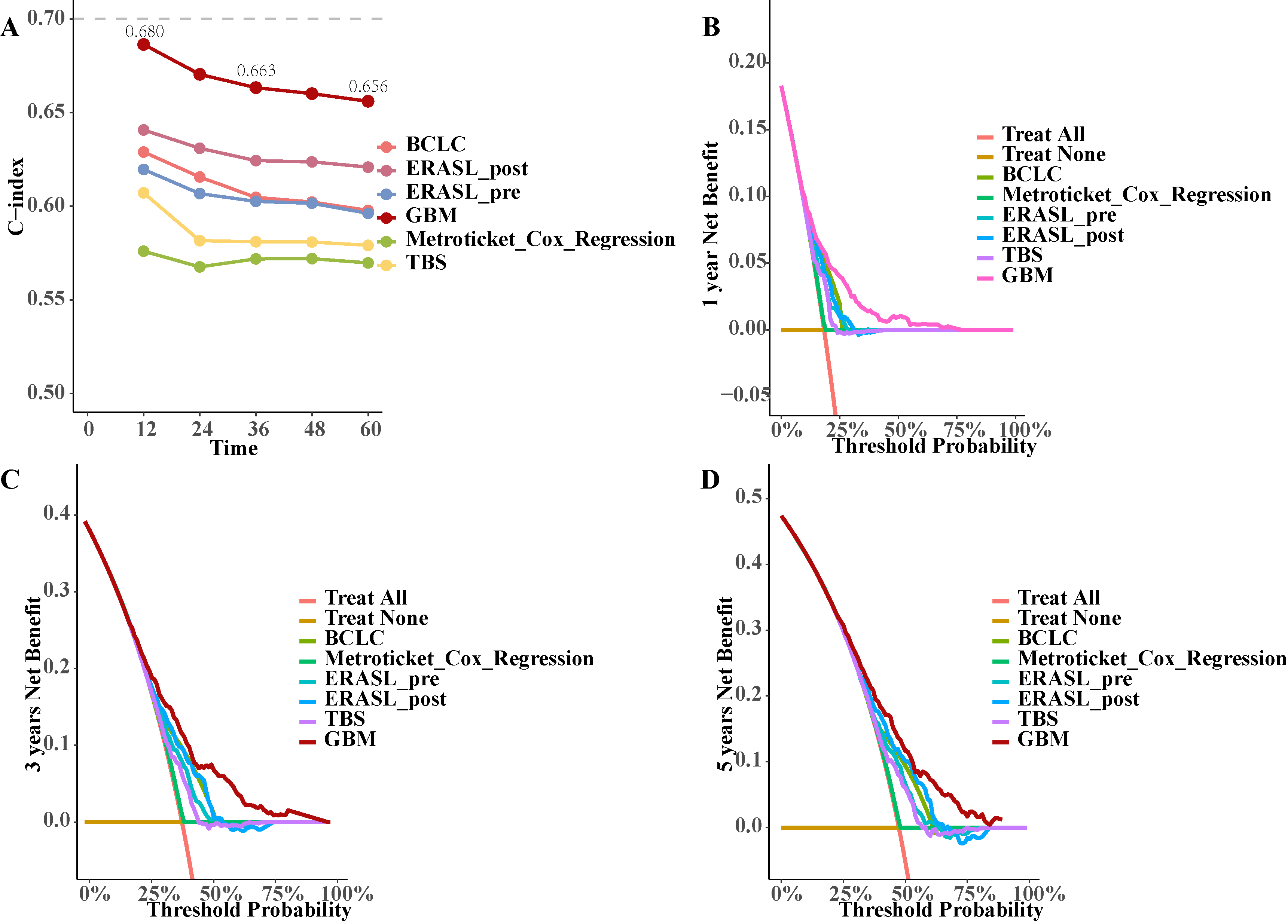

Supplement: Supplementary Figure 8 — Comparison of the predictive performance of the GBM model and previous prognostic models. (A) The C-index values of the GBM model and previous postoperative predictive models. (B-D) The 1-year, 3-year, and 5-year DCA curves of the GBM model and previous postoperative predictive models. [file Image8.tif]

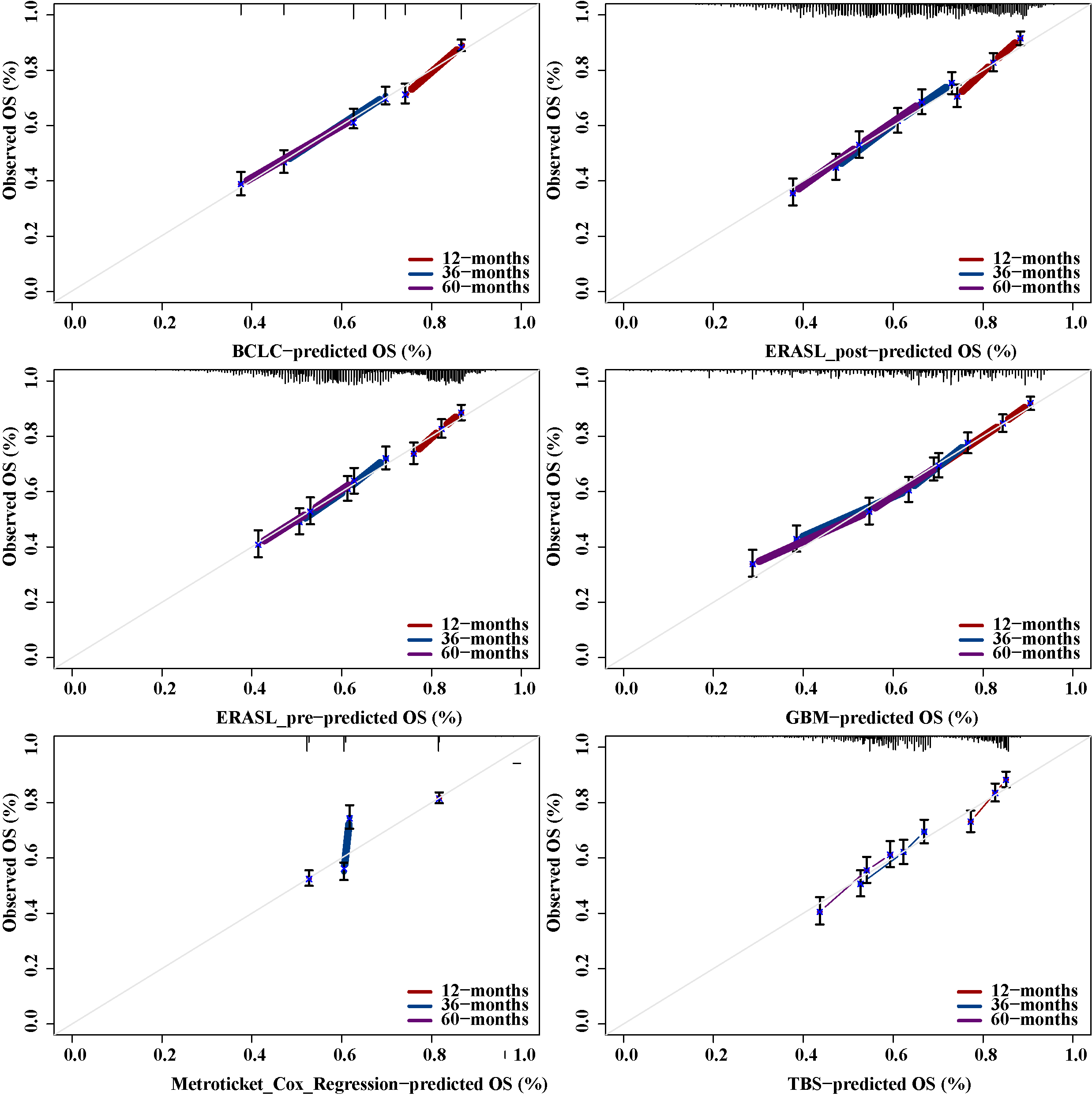

Supplement: Supplementary Figure 9 — The 1-, 3-, and 5-year calibration curves of the GBM model and previous postoperative predictive models. [file Image9.tif]
