# Supplementary material for: Development of a machine learning model to predict overall survival for large hepatocellular carcinoma at BCLC stage A or B after curative hepatectomy
Source: Front Immunol. 2025 Oct 21;16:1640075. doi: 10.3389/fimmu.2025.1640075 (PMC12583128; doi:10.3389/fimmu.2025.1640075)
Supplement: Supplementary Table 1 — The parameters of the machine learning algorithms. [file Table1.docx]

**Table S1. The parameters of the machine learning algorithms.**

| **Algorithms** | **Package** | **Core parameters** | **CV details** | **Details** |
| --- | --- | --- | --- | --- |
| Laasso_Cox | glmnet | family="cox", alpha=1, maxit=1000, seed=123 | Stratified 10-fold CV (stratified by OS status [1=death, 0=censored]); optimal lammda selected by minimizing partial likelihood deviance (lammda.min) + robustness check via lammda.1se (one-standard-error rule) | 10-fold CV via cv.glmnet; lammda.min=0.00396, lammda.1se=0.16358; lammda.min selected to retain 3 features (BCLC stage, MVI, tumor size); balances predictive performance and biological interpretability |
| GBM | Gbm | n.trees=1000, interaction.depth=5, n.minobsinnode=3, shrinkage=0.01, cv.folds=10, n.cores=6, seed=123 | Stratified 10-fold inner CV (stratified by OS status); grid search for hyperparameters; optimal combination selected by maximum C-index across folds | Grid search ranges: n.trees (500–2000), interaction.depth (3–7), shrinkage (0.001–0.05); early stopping to prevent overfitting; final model shows stable performance across CV folds |
| RSF | RandomForestSRC | ntree=1500, nodesize=5, mtry=2, splitrule="logrank", importance=TRUE, forest=TRUE, seed=123 | Stratified 10-fold inner CV (stratified by OS status); grid search for hyperparameters; optimal combination selected by maximum C-index across folds | Grid search ranges: ntree (1000–2000), nodesize (3–10), mtry (1–3); splitrule="logrank" for survival-oriented tree splitting; importance analysis confirms BCLC stage as top predictive feature |
| Coxboost | CoxBoost | penalty=optimized (start.penalty=50), maxstepno=25, K=3, type="naive", stepno=cv.res$optimal.step, seed=123 | 2-stage CV: ① Penalty optimized via optimCoxBoostPenalty with 3-fold CV (for computational efficiency); ② maxstepno tuned via 3-fold CV; both stratified by OS status; selected by minimum CV error | maxstepno tuned range (50–300); 3-fold CV used for penalty optimization to balance efficiency and robustness; "naive" CV type for independent fold partitioning |
| Survivalsvm | survivalsvm | gamma.mu=0.1, opt.meth="quadprog", diff.meth="makediff3", kernel="lin_kernel", sgf.sv=5, sigf=7, maxiter=100, margin=0.05, bound=10, seed=123 | Stratified 10-fold inner CV (stratified by OS status); grid search for key hyperparameters; optimal combination selected by maximum C-index across folds | Grid search ranges: gamma.mu (0.01–1), margin (0.01–0.1); "lin_kernel" used for linear feature relationships; maxiter=100 to ensure optimization convergence |
| xgboost | xgboost | objective="survival:cox", booster="gbtree", eval_metric="cox-nloglik", eta=0.03, max_depth=3, gamma=0.5, subsample=1, colsample_bytree=1, nrounds=100, early_stopping_rounds=50, seed=123 | Stratified 10-fold inner CV (stratified by OS status); grid search for hyperparameters; early stopping (50 rounds) to prevent overfitting; selected by minimum "cox-nloglik" across folds | Grid search ranges: eta (0.01–0.1), max_depth (2–6), gamma (0–1); eval_metric="cox-nloglik" for survival model fit; subsample=1 and colsample_bytree=1 to use full feature/sample information |
| superpc | superpc | type="survival", s0.perc=0.5, n.threshold=20, n.fold=10, n.components=3, min.features=3, compute.fullcv=TRUE, seed=123 | Stratified 10-fold CV (stratified by OS status); tuned s0.perc (0.3–0.7) and n.components (1–5); optimal parameters selected by maximum survival score (scor) across folds | "compute.fullcv=TRUE" for comprehensive fold-wise prediction; min.features=3 to ensure clinical interpretability; n.threshold=20 to explore optimal feature selection cutoff |
| plsRcox | plsRcox | nt=3, scale=TRUE, nfold=10, verbose=FALSE, seed=123 | Stratified 10-fold CV (stratified by OS status); tuned number of components (nt: 1–5); optimal nt selected by maximum C-index across folds | scale=TRUE for feature standardization (reduces multicollinearity); nt=3 balances model complexity and predictive performance; verbose=FALSE to avoid redundant output |

Abbreviations: Laasso_Cox: least absolute shrinkage and selection operator regression; GBM: gradient boosting machine; RSF: random survival forest; Coxboost: boosting for cox’s proportional hazards model; Survivalsvm: survival support vector machine; xgboost: extreme gradient boosting; superpc: super-predictor cox model; plsRcox: partial least squares with cox’s proportional hazards model, CV, cross-validation; C-index, concordance index.

In this study, the optimal lambda value for the LASSO-Cox model was determined through 10-fold cross-validation using the glmnet package in R. The cv.glmnet function was employed to rigorously evaluate model performance across different data subsets and mitigate the risk of overfitting. The selection of lambda was primarily based on minimizing the partial likelihood deviance (lambda.min), with additional robustness validation via the one-standard-error rule (lambda.1se), which identifies a more parsimonious model whose deviance remains within one standard error of the minimum. The cross-validation results showed that lambda.min was 0.00396 and lambda.1se was 0.16358. After comprehensive consideration, lambda.min was selected as the final lambda to retain more features with potential biological and predictive significance. The final LASSO-Cox model included three features: BCLC stage, MVI, and tumor size

**Table S2.** **Discriminative performance of eight ML models evaluated by C-index in stratified 10-Fold cross-validation and independent internal validation.**

| **Algorithms** | **C-index of 10-fold cross-validation on the training set** | **C-index of the independent internal validation set** | **Difference in C-index between cross-validation and independent validation** | **Relative difference (%)** |
| --- | --- | --- | --- | --- |
| RSF | 0.712 (0.037) | 0.664 | 0.048 | 6.74 |
| GBM | 0.694 (0.033) | 0.727 | 0.033 | 4.75 |
| Lasso-Cox | 0.62 (0.035) | 0.66 | 0.04 | 6.45 |
| CoxBoost | 0.64 (0.036) | 0.66 | 0.02 | 3.12 |
| Survivalsvm | 0.561 (0.058) | 0.585 | 0.024 | 4.28 |
| xgboost | 0.675 (0.033) | 0.653 | 0.022 | 3.26 |
| superpc | 0.566 (0.058) | 0.565 | 0.001 | 0.18 |
| plsRcox | 0.622 (0.033) | 0.67 | 0.048 | 7.72 |

Note: Values are presented as mean ±SD. Abbreviations: RSF: random survival forest; GBM: gradient boosting machine; Laasso_Cox: least absolute shrinkage and selection operator regression; Coxboost: boosting for cox’s proportional hazards model; Survivalsvm: survival support vector machine; xgboost: extreme gradient boosting; superpc: super-predictor cox model; plsRcox: partial least squares with cox’s proportional hazards model, CV, cross-validation; C-index, concordance index.

**Table S3. The Schoenfeld residual tests of the multivariate cox regression.**

| **Variables** | **chisq** | **df** | **p** |
| --- | --- | --- | --- |
| BCLC | 1.373272 | 1 | 0.241250489 |
| Gender | 2.813362 | 1 | 0.093482287 |
| Age | 0.319448 | 1 | 0.571939608 |
| BMI | 0.305583 | 1 | 0.580403438 |
| Hepertension | 0.078482 | 1 | 0.779364541 |
| Diabetes | 0.780078 | 1 | 0.377117184 |
| Smoke | 0.314813 | 1 | 0.574741865 |
| Family history | 0.211248 | 1 | 0.645790747 |
| HBsAg | 0.092914 | 1 | 0.760504708 |
| HBeAg | 1.356659 | 1 | 0.244117428 |
| HBV DNA | 0.000887 | 1 | 0.976236138 |
| HCV | 0.162781 | 1 | 0.686609186 |
| Number of tumors | 0.320285 | 1 | 0.571436144 |
| Microvascular invasion | 0.110835 | 1 | 0.739195009 |
| Child-Pugh stage | 1.388435 | 1 | 0.238669524 |
| Total bilirubin | 2.485309 | 1 | 0.114913782 |
| Albumin | 0.118941 | 1 | 0.730186069 |
| Pre-albumin | 0.307347 | 1 | 0.579312994 |
| Alanine transaminase | 4.98738 | 1 | 0.025532842 |
| Aspartate aminotransferase | 2.059428 | 1 | 0.151266908 |
| Alpha-fetoprotein | 0.831359 | 1 | 0.361879719 |
| CA19_9 | 0.357667 | 1 | 0.549804763 |
| Prothrombin time | 3.270998 | 1 | 0.070514697 |
| Platelets | 0.898714 | 1 | 0.343126842 |
| Absolute value of white blood cell | 0.551615 | 1 | 0.457658431 |
| Absolute value of lymphocyte | 1.453083 | 1 | 0.228033833 |
| NLR | 0.453513 | 1 | 0.500671154 |
| Tumor size | 2.363575 | 1 | 0.124197261 |
| GLOBAL | 29.76801 | 28 | 0.439153375 |

Abbreviations: BMI: body mass index; BCLC: Barcelona Clinic Liver Cancer staging system; NLR: neutrophil - lymphocyte ratio.

**Table S4. Univariable and multivariable Cox regression analysis of factors in the training group.**

| Factors | Univariate Analysis | | | Multivariate Analysis | |
| --- | --- | --- | --- | --- | --- |
|  | (HR, 95%CI) | P | P_FDR | （HR, 95%CI) | P |
| BCLC (B) | 2.07 (1.73-2.48) | <0.001 | <0.001 | 1.87 (1.48-2.36) | <0.001 |
| Gender (Male) | 1.17 (0.88-1.56) | 0.265 | 0.513 |  |  |
| Age (≥65, years) | 1.00 (0.80-1.25) | 0.992 | 0.992 |  |  |
| BMI (≥24, kg/m^2^) | 0.97 (0.70-1.33) | 0.837 | 0.962 |  |  |
| Hypertension (Presence) | 0.98 (0.74-1.30) | 0.888 | 0.962 |  |  |
| Diabetes (Presence) | 1.02 (0.75-1.40) | 0.896 | 0.962 |  |  |
| Smoke (Presence) | 1.18 (0.99-1.41) | 0.069 | 0.199 |  |  |
| Family history (Presence) | 1.06 (0.84-1.32) | 0.627 | 0.791 |  |  |
| HBsAg (Positive) | 0.94 (0.74-1.18) | 0.585 | 0.772 |  |  |
| HBeAg (Positive) | 1.15 (0.96-1.37) | 0.137 | 0.334 |  |  |
| HBV DNA (≥500) | 1.15 (0.96-1.38) | 0.138 | 0.334 |  |  |
| HCV (Presence) | 0.67 (0.21-2.08) | 0.488 | 0.674 |  |  |
| Number of tumors (Multiple) | 1.61 (1.33-1.94) | <0.001 | <0.001 | 1.02 (0.80-1.29) | 0.885 |
| Microvascular invasion (Presence) | 1.79 (1.49-2.15) | <0.001 | <0.001 | 1.55 (1.29-1.88) | <0.001 |
| Child-Pugh stage (B) | 1.21 (0.87-1.68) | 0.247 | 0.512 |  |  |
| Total bilirubin (>17.1μmol/L) | 0.87 (0.63-1.19) | 0.369 | 0.564 |  |  |
| Albumin (≥35g/L) | 0.90 (0.73-1.10) | 0.314 | 0.536 |  |  |
| Pre-albumin (≥200mg/L) | 0.81 (0.67-0.98) | 0.03 | 0.098 |  |  |
| Alanine transaminase (≥40U/L) | 0.99 (0.83-1.19) | 0.95 | 0.984 |  |  |
| Aspartate aminotransferase (≥40U/L) | 1.25 (1.04-1.51) | 0.018 | 0.074 |  |  |
| Alpha-fetoprotein (≥400ng/mL) | 1.25 (1.04-1.49) | 0.015 | 0.071 |  |  |
| CA19_9 (>37KU/L) | 1.04 (0.79-1.37) | 0.786 | 0.95 |  |  |
| Prothrombin time (≥13s) | 1.08 (0.90-1.29) | 0.43 | 0.623 |  |  |
| Platelets (≥300, 10^9^/L) | 1.12 (0.88-1.43) | 0.367 | 0.564 |  |  |
| Absolute value of white blood cell (10^9^/L) | 1.05 (1.01-1.09) | 0.028 | 0.098 |  |  |
| Absolute value of lymphocyte (10^9^/L) | 1.02 (0.98-1.06) | 0.244 | 0.512 |  |  |
| NLR (<5) | 0.84 (0.61-1.16) | 0.296 | 0.536 |  |  |
| Tumor size (cm) | 1.06 (1.04-1.09) | <0.001 | <0.001 | 1.04 (1.01-1.07) | 0.005 |

Abbreviations: HR, hazard ratio; CI, confidence interval. BMI: body mass index; BCLC: Barcelona Clinic Liver Cancer staging system; NLR: neutrophil - lymphocyte ratio. P_FDR: p-values was adjusted using the False Discovery Rate (FDR) correction via the Benjamini-Hochberg method.

**Table S5** **IBS in training and test sets and NRI at 1-, 3-, and 5-year follow-up for different ML models**

| **Model** | **Trainset IBS** | **Testset IBS** | **1 year NRI** | **3 years NRI** | **5 years NRI** |
| --- | --- | --- | --- | --- | --- |
| Coxboost | 0.2119 | 0.2097 | 0.2462 | 0.256 | 0.2576 |
| xgboost | 0.2441 | 0.2488 | 0.2977 | 0.3172 | 0.3151 |
| GBM | 0.2663 | 0.2723 | 0.3284 | 0.3274 | 0.3391 |
| plsRcox | 0.3573 | 0.3479 | 0.2499 | 0.2653 | 0.2672 |
| superPC | 0.3908 | 0.3844 | 0.1709 | 0.1597 | 0.1734 |
| LASSO-Cox | 0.5326 | 0.5259 | 0.2499 | 0.2653 | 0.2672 |
| RSF | 0.8764 | 0.8747 | 0.3691 | 0.4147 | 0.4405 |
| Survivalsvm | 0.8765 | 0.8747 | 0.1415 | 0.1138 | 0.1269 |

Abbreviations: RSF: random survival forest; GBM: gradient boosting machine; Laasso_Cox: least absolute shrinkage and selection operator regression; Coxboost: boosting for cox’s proportional hazards model; Survivalsvm: survival support vector machine; xgboost: extreme gradient boosting; superpc: super-predictor cox model; plsRcox: partial least squares with cox’s proportional hazards model. IBS: integrated brier score; NRI: net reclassification index.

**Table S6. Predictive performance of the previous postoperative predictive models.**

| Models | 1-year AUC | 3-year AUC | 5-year AUC | 1-year C-index | 3-year C-index | 5-year C-index |
| --- | --- | --- | --- | --- | --- | --- |
| GBM | 0.714 (0.679-0.749) | 0.708 (0.679-0.738) | 0.705 (0.674-0.736) | 0.686 | 0.663 | 0.656 |
| ERASL-post | 0.662 (0.627-0.697) | 0.663 (0.632-0.694) | 0.660 (0.627-0.693) | 0.641 | 0.624 | 0.621 |
| ERASL-pre | 0.635 (0.599-0.671) | 0.628 (0.597-0.660) | 0.614 (0.580-0.648) | 0.620 | 0.603 | 0.596 |
| BCLC | 0.644 (0.613-0.676) | 0.621 (0.593-0.648) | 0.608 (0.579-0.637) | 0.629 | 0.605 | 0.598 |
| TBS | 0.622 (0.586-0.657) | 0.610 (0.576-0.644) | 0.609 (0.577-0.641) | 0.607 | 0.581 | 0.579 |
| Metroticket cox regression | 0.580 (0.555-0.606) | 0.592 (0.567-0.617) | 0.591 (0.563-0.619) | 0.576 | 0.572 | 0.570 |

Note: Values are presented as means (95% confidence interval). Abbreviations: AUC: area under the receiver operating characteristic curve; C-index: concordance index; GBM: gradient boosting machine. ERASL-post: ERASL-post score; ERASL-pre: ERASL-pre score; BCLC: Barcelona Clinic Liver Cancer staging system; TBS: tumor-burden score.
